# A Model-Based Approach to Identify Binding Sites in CLIP-Seq Data

**DOI:** 10.1371/journal.pone.0093248

**Published:** 2014-04-08

**Authors:** Tao Wang, Beibei Chen, MinSoo Kim, Yang Xie, Guanghua Xiao

**Affiliations:** 1 Quantitative Biomedical Research Center, Department of Clinical Sciences, University of Texas Southwestern Medical Center, Dallas, Texas, United States of America; 2 Simmons Cancer Center, University of Texas Southwestern Medical Center, Dallas, Texas, United States of America; CSIR-Institute of Microbial Technology, India

## Abstract

Cross-linking immunoprecipitation coupled with high-throughput sequencing (CLIP-Seq) has made it possible to identify the targeting sites of RNA-binding proteins in various cell culture systems and tissue types on a genome-wide scale. Here we present a novel model-based approach (MiClip) to identify high-confidence protein-RNA binding sites from CLIP-seq datasets. This approach assigns a probability score for each potential binding site to help prioritize subsequent validation experiments. The MiClip algorithm has been tested in both HITS-CLIP and PAR-CLIP datasets. In the HITS-CLIP dataset, the signal/noise ratios of miRNA seed motif enrichment produced by the MiClip approach are between 17% and 301% higher than those by the *ad hoc* method for the top 10 most enriched miRNAs. In the PAR-CLIP dataset, the MiClip approach can identify ∼50% more validated binding targets than the original *ad hoc* method and two recently published methods. To facilitate the application of the algorithm, we have released an R package, *MiClip (* http://cran.r-project.org/web/packages/MiClip/index.html *)*, and a public web-based graphical user interface software (http://galaxy.qbrc.org/tool_runner?tool_id=mi_clip) for customized analysis.

## Introduction

RNA-binding proteins (RBPs) participate in RNA translation, splicing and editing events, and play essential roles in mRNA maturation and downstream regulation of cellular events [1]. Hundreds of RBPs are encoded by the vertebrate genomes, and each RBP has its specific RNA binding properties. For example, AGO protein is an RBP that serves as a platform of mRNA and small RNA interactions [2], and the FET family RBPs, including FUS, EWSR1 and TAF15, play important roles in RNA editing and human cancers [3], [4]. Thus, accurate identification of RBP binding targets is important to a systematic understanding of transcription, translation and other biological processes within cells.

Cross-linking immunoprecipitation coupled with high-throughput sequencing (CLIP-Seq) technique has been developed to study genome-wide RNA-protein interactions [5]–[7]. The general procedures of CLIP include covalently linking RNA with RBP, isolating the bound complex, removing the protein and converting RNA to cDNA for sequencing. The CLIP experiments evolved quickly with the development of next generation sequencing (NGS) techniques. The most commonly used CLIP-Seq experiments are: 1. high-throughput sequencing of RNA isolated by cross-linking immunoprecipitation (HITS-CLIP) [5], [8], [9], and 2. photoactivatable-ribonucleoside-enhanced cross-linking and immunoprecipitation (PAR-CLIP) [10]–[12]. Depending on the cell culture and cross-linking method being used, various types of sequencing errors are introduced at RBP binding sites with certain probabilities [8]. HITS-CLIP utilizes UV cross-linking of proteins with RNA and introduces mutations in the sequencing data. More specifically, the mutations are induced on the cDNAs generated in the reverse transcription step from the RNA fragments when the reverse transcription enzyme incorporates an incorrect nucleotide at the site of the cross-linked nucleotide due to attachment of the remaining residues of the covalently bound protein. However, the type of mutations is not well-defined and may vary for different proteins [8], [13]. PAR-CLIP utilizes photoreactive ribonucleoside analogs for incorporation into RNA and some of the analogs that are cross-linked by UV treatment at a later step will result in specific nucleotide substitution events. For example, 4-thiouridine treatment will induce T->C mutations at crosslinking sites [12]. The iCLIP experiment, which involves a self-circularization step to capture the truncation of cDNA reads, is less commonly used than HITS-CLIP and PAR-CLIP [14]. In iCLIP experiments, cDNA counts, rather than total tag count and mutant tag count, are used to infer protein binding sites. The analysis of iCLIP experiments requires distinct procedures and is therefore not considered in the *MiClip* package.

The CLIP-Seq techniques allow genome-wide mapping of RNA-protein interactions, but they also present computational challenges for identifying the true binding sites from millions of short reads with mutation information. In most studies, *ad hoc* methods are employed to process CLIP-Seq data. Generally, CLIP clusters are formed by overlapping the short sequencing tags and simple cut-offs are applied to produce lists of reliable CLIP clusters [3], [5], [9]. However, these methods are sensitive to the choice of cut-off values, and there are no confidence values associated with the identified binding sites. To better analyze CLIP-Seq datasets, a few bioinformatics tools have been developed recently. CLIPZ is an online server for analyzing CLIP-Seq datasets [15]. However, it still works under an *ad hoc* framework — no significant levels are given for the resulting binding sites. *wavClusteR* [16] is designed to analyze PAR-CLIP experiments. It assumes a two-component mixture model based on relative substitution frequencies (RSFs) for identifying reliable binding sites, and employs wavelet transformation for resolving peak boundaries. *PARalyzer* [17] is also designed to analyze PAR-CLIP experiments. *PARalyzer* identifies reliable binding sites as nucleotides with a minimum read depth and having a higher likelihood of T->C conversion than non-conversion. *PARalyzer* and *wavClusteR* could not be easily extended to other types of CLIP-seq datasets due to the underlying model assumptions. Besides, StarBase v2.0 [18] is a comprehensive database with more than 100 CLIP-Seq datasets.

Here, we present a model-based approach, MiClip, for analyzing both HITS-CLIP and PAR-CLIP data. This approach has been implemented in the R statistical environment [19]. It first removes duplicates and finds CLIP clusters, then divides the task of identifying reliable binding sites into two rounds of Hidden Markov Model (HMM). The first HMM infers enriched *vs.* non-enriched regions in CLIP clusters, and the second HMM infers binding sites of RBPs *vs.* non-binding sites within the enriched regions. Finally, the reliable binding sites and the CLIP clusters containing these sites are reported in a user-friendly format. We have tested this algorithm on two datasets and shown that MiClip provides a general and efficient framework for identifying high-confidence RBP binding sites at high resolution.

## Materials and Methods

### CLIP-Seq datasets and mapping

The AGO HITS-CLIP dataset described in Chi, *et al.* [9] was downloaded from http://AGO.rockefeller.edu. The alignment of the HITS-CLIP data was done by Novoalign (*Novocraft Technologies*) to the mm10 reference genome. After alignment, the resulting SAM files from Brain A–E were pooled in the HITS-CLIP dataset. The FET PAR-CLIP data described in Hoell, *et al.* [3] was downloaded from DRASearch, with study number SRP003889. This dataset contains the PAR-CLIP experiments of three proteins, TAF15, EWSR1 and FUS, in both inducible and stable forms. The PAR-CLIP datasets were also aligned by Novoalign to the hg19 genome using parameters. Multiple-hit reads are not reported for both datasets. The resulting SAM files of both the inducible and stable experiments for the same protein were pooled in the PAR-CLIP dataset.

### Finding CLIP clusters by overlapping CLIP-Seq tags

The MiClip package took the alignment SAM format files as input. In each dataset, duplicate reads that have the same mapping coordinates (including strand) were identified and collapsed to a single “tag”. Tags overlapping by at least one nucleotide were grouped together to form CLIP clusters, and those not overlapping with any other tags were discarded. The deletions on each base were counted as mutation events for the AGO dataset, and the T->C substitutions on each base on the correct strand were counted as mutation events for the TAF15, EWSR1 and FUS datasets.

### Identify enriched regions (first round HMM)

To identify enriched regions, CLIP clusters were divided into bins of 5 bp. Let 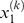 be the total tag count in the *t*-th bin of *k*-th cluster, so cluster *k* could be represented as a series of tag count numbers: 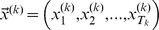. Here we used HMM to determine the enriched regions from observed tag counts.

**Figure 1:**
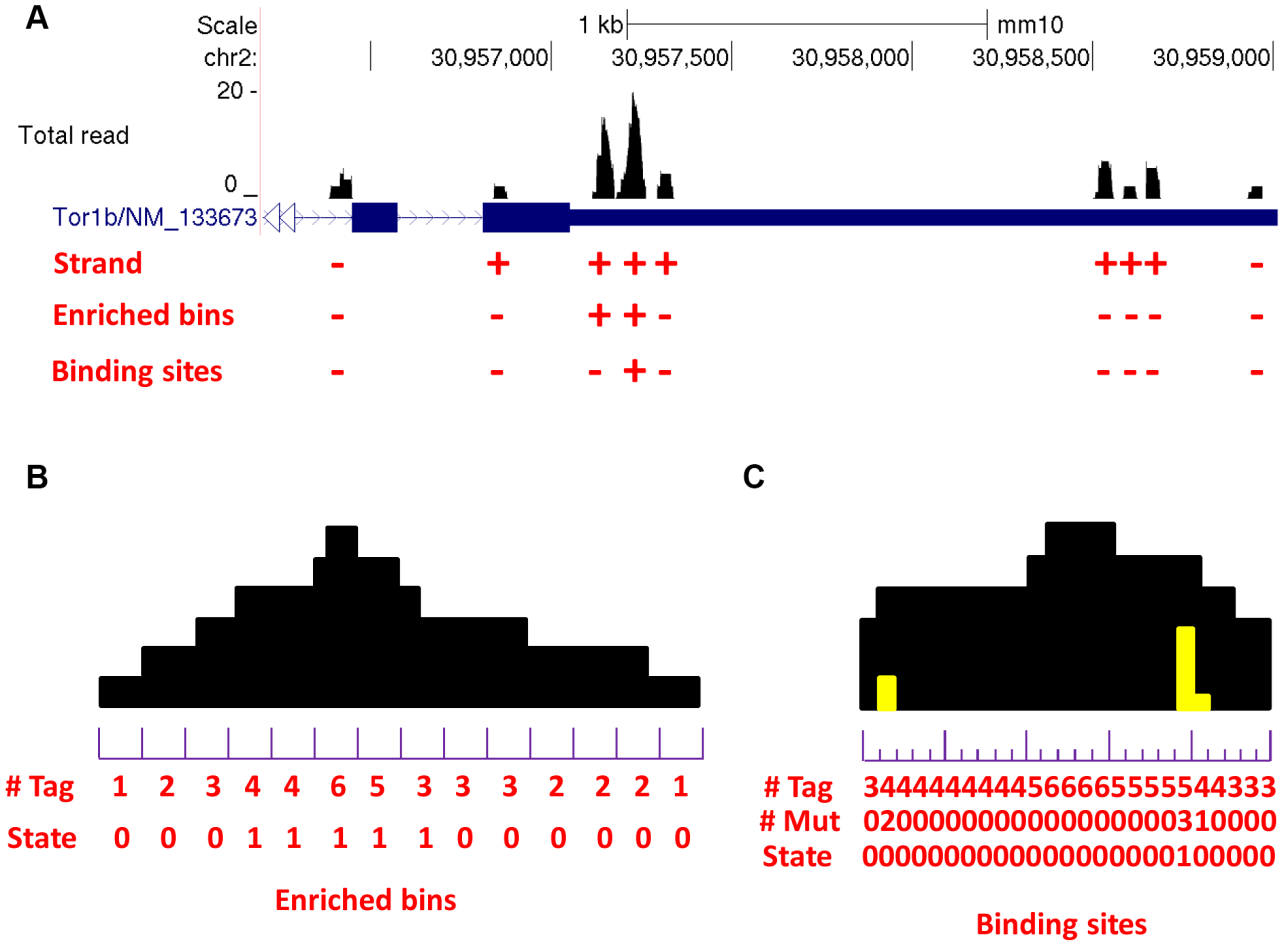
Demonstration of the MiClip algorithm. (a) The inference results of MiClip on AGO CLIP clusters. At the bottom, “Strand” refers to the strands of clusters, “Enriched bins” shows whether a cluster contains enriched bins and “Binding sites” shows whether a cluster contains binding sites. This region covers part of the Tor1b gene. (b) A cartoon of the first HMM. At the bottom, “# Tag” refers to the rounded number of tags covering each bin and “State” shows whether a bin was inferred to be “enriched”. (c) A cartoon of the second HMM. At the bottom, “# Tag” refers to the number of total tags covering each base, “# Mut” refers to the number of mutant tags covering each base and “State” shows whether a base was inferred to be a “binding site”.

The HMM has two states: 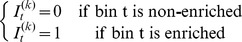. Poisson model is a popular model to fit the count data [20], [21]. Given state 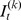, the observed tag counts were modeled by a two-component Poisson mixture model: 
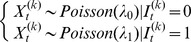
, so the emission probability could be written as 
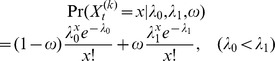
, where the ω is the proportion of enriched bins in the CLIP clusters. The transition matrix Π is a 2×2 matrix, where element 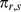 is the transition probability 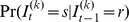. We estimated the λ_0_, λ_1_ and ω parameters first from the observed data using method of moments [22], and then used the standard Viterbi algorithm [23] to infer the hidden states 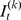, namely the enriched *vs.* non-enriched bins. The Viterbi algorithm determines the hidden state of each bin according to the criterion that posterior probability of each bin in the inferred state (enriched or non-enriched) should be larger than the posterior probability of this bin in the other state, given the model and observation. Finally, the adjacent enriched bins were concatenated into enriched regions. A cartoon illustration of how first round HMM works is shown in **Fig. 1b**.

### Identify reliable binding sites (second round HMM)

To identify reliable binding sites, the enriched regions were further divided into bins of 1 bp. Let 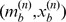 be the number of mutations and total tag count in the *b*-th base pair of the *n*-th enriched region. This HMM was designed to have two states: 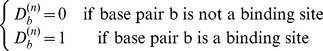 The observed number of mutations 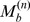 given the tag count 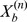 given 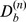 was modeled by 
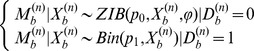
, here a zero inflated binomial distribution (ZIB) [24] with probability 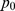, size 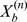 and inflation parameter φ was used to model the background mutations, such as random sequencing errors at non-binding sites (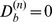), and a binomial distribution with probability 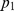 and size 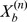 was used to model the cross-linking induced mutations at RBP binding sites (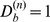). So, the emission probability could be written as: 
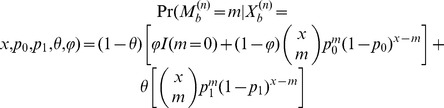
 where θ is the proportion of binding sites in enriched regions. We estimated the parameters as follows: First, from the density plot of mutation rates *(m/x)*, assume that we could observe two modes 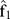 and 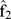, where 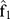 corresponds to the probability for success of the background ZIB component and 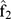 corresponds to the probability of success for the binomial component. Then, we chose a parameter *c* specified by the user, so that 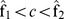. The bins with mutation ratio 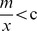 were used to estimate 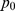 and φ for ZIB distribution using method of moments, and the remaining bins were used to estimate 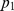 for the binomial distribution. According to our simulation studies (data not shown), the estimation procedure is robust and the choice of parameter c will not greatly change the estimated parameters. Again, the standard Viterbi algorithm [23] was used to infer the hidden states, and the probability of being a reliable binding site 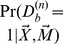 was calculated for each base pair *b*. The enriched part of the peak in **Fig. 1b** is shown as a cartoon in **Fig. 1c** to illustrate how second round HMM works.

### Genome Browser visualization

For all CLIP clusters, only the mapped tags that comprise each cluster were taken into account when calculating base coverage. Then, a BedGraph format file was generated for each sample, and uploaded onto UCSC Genome Browser for visualization.

### Motif Analysis

Exact matches to the 7-mer seed motifs of the top 10 most enriched miRNAs were scanned by an in-house Perl script (this script was included in the exec folder of the *MiClip* package for users to replicate our results). The Perl script scanned the 7-mers through the genomic sequences covered by all significant clusters in the HITS-CLIP dataset and reported the locations of matches. To estimate the signal to noise ratio, 40,000 background sequences (with the same length as the mean length of the identified clusters) that have no overlapping regions with the target sequences were randomly chosen from the mouse genome. The same scanning procedure was done in these background sequences to calculate the signal/noise ratio for miRNA seed motif enrichment. The relative distances from the binding site to the centers of the matches within each cluster were calculated. If a cluster has more than one possible binding site, the shortest distance was kept for analysis.

### RNA structure prediction

The RNA sequence of *SNORD58C* on the negative strand was fetched from UCSC Genome Browser. The structure of *SNORD58C* snoRNA was predicted by the RNAfold online server (http://rna.tbi.univie.ac.at/cgi-bin/RNAfold.cgi), which predicts RNA structure by minimum free energy (MFE) and partition function. All default options provided by the server were used.

## Results

### AGO HITS-CLIP dataset

We focus the demonstration of the MiClip method on the AGO HITS-CLIP dataset described in Chi, *et al.* [9], while for the FET PAR-CLIP dataset we will only present the results. In the AGO HITS-CLIP study, AGO protein bound to mouse brain RNAs was purified by UV-irradiating P13 neocortex and immunoprecipitation. After purification, complexes of two different modal sizes were observed: 110 kD complexes harboring miRNAs (miRNA library) and 130 kD complexes harboring mainly mRNAs (mRNA library). The MiClip analysis was carried out only on the mRNA library. All five replicates of the AGO mRNA datasets were pooled, resulting in a total of around 26 million reads. Around 22 million reads were aligned successfully to the mm10 reference genome by Novoalign. Removing duplicates has been shown to be important in CLIP-Seq analysis, and many recent studies adopt slightly different methods of collapsing duplicate reads in specific datasets [25]–[27]. To be general, MiClip reads with exactly the same chromosome, strand, start site and end site were defined as duplicates and collapsed to one tag. About 1.6 million unique tags were kept after removing duplicates. Then, tags overlapping by at least one nucleotide were grouped together to form CLIP clusters. Around 380,000 clusters with two or more overlapping tags were formed. According to the study by Zhang, *et al.* [8], deletion is the characteristic marker mutation for protein-mRNA interaction sites in AGO and Nova HITS-CLIP experiments. Thus, we only counted the occurrences of deletions on each genomic site in our analysis for this dataset.

To identify the enriched regions, all clusters were divided into bins of 5 bp, resulting in a total of 3,525,678 bins. On average, each cluster was divided into ∼9 bins. All the bins derived from the same cluster were defined as one observation sequence. A two-component-Poisson mixture model was fitted for all the tag counts on the bin-level, and the status of being enriched or non-enriched for each bin was inferred from the first round HMM. After the first round HMM, 291,160 enriched bins were identified, which correspond to 39,471 clusters. The distributions of tag counts of enriched *vs.* non-enriched bins were shown in **Fig. S1a** in **File S1**. Then, adjacent enriched bins were concatenated together and formed 41,078 enriched regions. This number is larger than 39,471, because some clusters have multi-modal peaks, which results in multiple enriched regions inside one cluster. One advantage of the HMM in this step is that it could discriminate CLIP clusters with different “shapes”. For example, a “flat” cluster (**Fig. S2** in **File S1**) that is comprised of 14 unique tags and spans 41 bins was not classified as enriched, while a cluster with a similar number of tags, but “sharper” pileup, would be more likely to be deemed as enriched.

To identify reliable binding sites, total tag and mutant tag information was collected on a single nucleotide basis within the enriched regions, resulting in 1,441,030 bases. Then, a mixture model of a zero-inflated binomial distribution and a binomial distribution was fitted for the total tag numbers and mutant tag numbers of all 1,441,030 bases. The zero-inflated binomial distribution was used to encompass background mismatches on non-binding sites, such as random PCR and sequencing errors, and the binomial distribution was used to encompass mismatches induced by cross-linking on binding sites. The status of binding and non-binding at each base pair was then inferred by using the second round HMM, and 6,867 single-nucleotide binding sites were identified. There are 5,795 out of all 39,471 enriched CLIP clusters containing binding sites, and most of them contain only one binding site per cluster (**Table S1** in **File S1**). The distribution of mutation proportion of binding sites *vs.* non-binding sites is shown in **Fig. S1b** in **File S1**. We also randomly permuted the total tag counts and mutant tag counts data, and the *MiClip* algorithm found a total of 665 CLIP clusters with at least one reliable binding site in the permuted data. Therefore, the False Discovery Rate (FDR) is 0.11.

**Figure 2:**
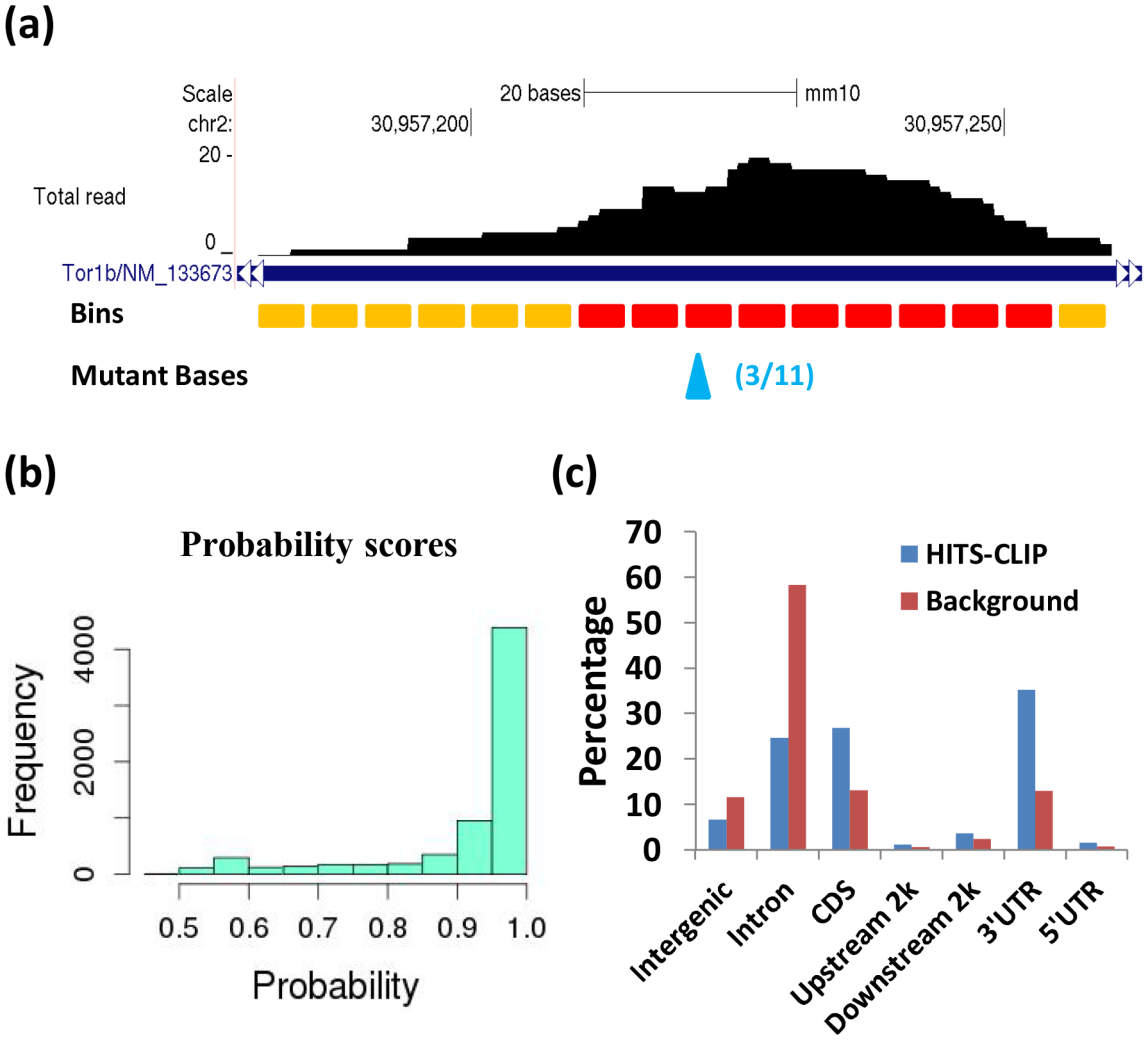
The results produced by MiClip on the HITS-CLIP dataset. (a) One exemplary cluster with an identified binding site. The Genome Browser visualization shows the tag pileup of this cluster. Orange bars denote non-enriched bins, red bars denote enriched bins and the blue arrow points to the binding site. The number of mutant tags and the total tag count at this base are shown in the parentheses. (b) Histogram of the probability scores of all 6,867 binding sites. The x-axis is the probability of a site being a binding site and the y-axis is the frequency. (c) The percentages of all 5,795 reliable clusters identified by *MiClip* and all reads as background control mapped to each annotation category. CDS represents coding sequence, upstream 2k represents 2 kb regions upstream of the 5′UTRs, downstream 2k means 2 kb regions downstream of 3′UTRs and Intergenic represents distal genomic regions not included in all the other categories.

All CLIP clusters were marked as to whether they contain enriched regions, and for clusters with enriched regions they were marked as to whether they contain at least one binding site (**Fig. 1a**). A CLIP cluster containing at least one enriched bin with binding site(s) was reported as a reliable cluster. One exemplary cluster (the fourth cluster in **Fig. 1a**) with an identified binding site was shown in more detail in **Fig. 2a**. The orange bars show non-enriched bins, the red bars show enriched bins and the blue arrow points to the binding site. For each identified binding site, MiClip produced a probability score (**Fig. 2b**), which could be used to prioritize subsequent validation experiments. A site with a higher probability score means this site is a more reliable binding site. When we aligned the reliable clusters to the mouse genome and summarized the genomic locations of these clusters (**Fig. 2c**), we discovered that the largest portion of the clusters (35%) falls into 3′UTR. This is followed by coding sequences (27%) and intronic regions (25%), while 5′UTR, 5′UTR extended regions and 3′UTR extended regions each contain 1.6%, 1.2% and 3.6%, respectively, of the clusters. Within mRNAs, the clusters are highly enriched in 3′UTR (∼55%). This observation is consistent with previous knowledge of miRNA regulation [28]. Also the percentage of each annotation type is distinctly different from the background distribution of the annotation types of all the reads as control (**Fig. 2c**), supporting the algorithm's ability of filtering for true binding sites in the 3′UTR. Therefore our results suggest that the MiClip method is able to find reliable CLIP clusters with functional significance.

**Figure 3:**
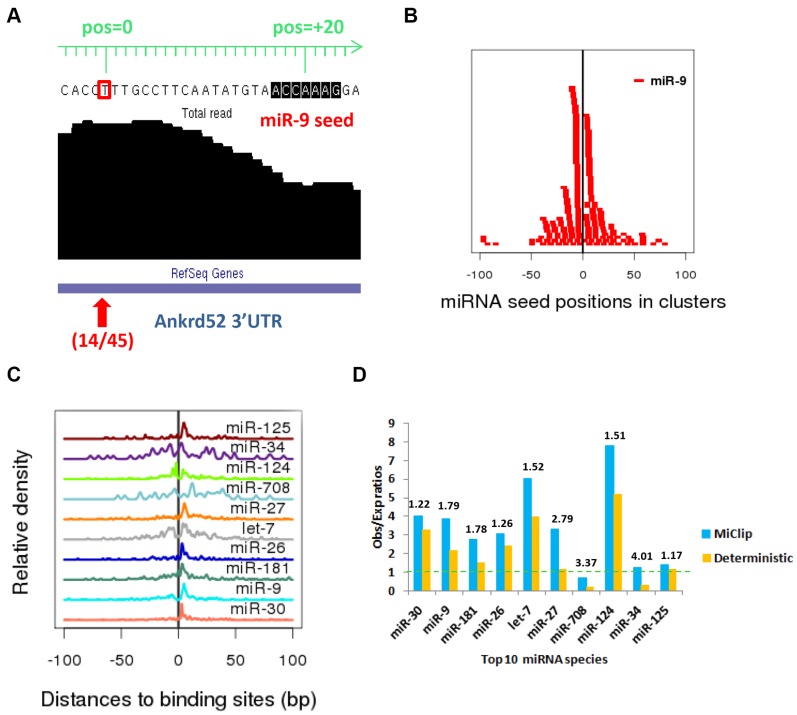
miRNA seed matches in AGO mRNA CLIP clusters. (a) An exemplary cluster with one reliable binding site and a seed match for miR-9. The red rectangle is the binding site, which has 14 mutant tags out of 45 total tags. The miR-9 seed motif is ACCAAAG, which locates 20 bp downstream of the binding site. (b) The positions of seed matches of miR-9 (positions 2–8) within the 5,795 significant clusters. 4 out of 199 seed motifs are more than 100 bp upstream or downstream away from the binding sites, which are ignored. X-axis is the relative position with respect to the binding site within each cluster. (c) The pileup of seed matches of the top 10 miRNAs (positions 2–8) within the 5,795 significant clusters drawn as density curves. 49 out of 1557 seed motifs (all top 10 miRNAs) are more than 100 bp upstream or downstream away from the binding sites, which are ignored. (d) The signal/noise ratio of miRNA seed motif enrichment. The blue bars show enrichment ratios for MiClip and the orange bars show enrichment ratios for the *ad hoc* method. The numbers on top of these bars show the signal/noise ratio for clusters found by MiClip divided by the signal/noise ratio for clusters found by the *ad hoc* method.

To further validate the MiClip approach, we tried to correlate identified CLIP mRNA clusters with miRNA seed matches. A recent X-ray study suggests that the AGO proteins function by forming ternary structures with miRNA and mRNA [29]. By mapping short reads from the miRNA library, Chi, *et al.* (Chi et al. 2009) were able to identify the most enriched miRNA species and rank the miRNA species by their abundance, with the most abundant miRNA being miR-30. We scanned for the 7-mer (position 2–8) seed motif matches for the top ten most enriched miRNAs within the 5,795 clusters. **Fig. 3a** shows an exemplary significant CLIP cluster with a motif match for miR-9. The red arrow points to the identified binding site, which has 14 deletions, and the probability of this site being a true binding site is >0.999. A miR-9 seed motif match occurs at 20 bp away downstream of the binding site. We calculated the relative distances to the centers of the miR-9 seed motifs from the binding sites within all reliable clusters containing miR-9 motifs. For the example shown in **Fig. 3a**, this distance is +20. Then, we plotted the positions of conserved miR-9 seed matches relative to binding sites according to the calculated distances (**Fig. 3b**). We found that most of the miR-9 seed matches are within −50 to +50 bp of binding sites and form a sharp peak around position 0, which are the binding sites. Also, we plotted the distances relative to the binding sites for all the top 10 miRNAs in **Fig. 3c**, and in this figure we plotted the pileup of seed motifs as density curves, for the sake of clarity. The seed matches for all top 10 miRNA motifs also tend to form very sharp peaks towards position 0, confirming the validity of the identified clusters and binding sites. Interestingly, the vertical line at pos = 0 does not cross many motif matches in **Fig. 3b** and **Fig. 3c**, indicating the binding sites are not located directly within seed motifs. This is because in the AGO HITS-CLIP experiment, miRNA and mRNA were paired at the seed motif, and thus partially protected from cross-linking. Similar phenomena were observed in previous work by another group [17]. Another interesting point to note is that seed motifs of some miRNAs, like miR-9, are predominantly downstream of binding sites, while motifs of other miRNAs, like let-7, locate both upstream and downstream of binding sites in large numbers. Overall, these results confirm the validity of the identified binding sites by MiClip, and show that using binding sites rather than peak summits, as in the original study, is more informative for finding protein binding locations.

Moreover, we calculated the percentages of the 5,795 clusters with seed motif matches for the top 10 miRNAs, as well as the percentages in 40,000 background sequences, for computing the signal/noise ratios for the top 10 miRNAs, as in the original publication [9] and others [30] in the field of nucleotide motif discovery. We found that 9 out of 10 miRNA seed matches are enriched (**Table 1**). In the original publication, the authors also provided enrichment ratios for the top 10 miRNAs from their list of significant clusters, but the enrichment ratios are not as high as calculated from the results of MiClip (**Fig. 3d**). For each miRNA, signal/noise ratio by the MiClip method over the *ad hoc* method ranges from 1.17 for miR-125 to 4.01 for miR-34 (**Fig. 3d**). In conclusion, MiClip is able to find AGO binding sites and reliable CLIP clusters with better biological significance than the *ad hoc* method used in the original work.

**Table 1:** The enrichment of the top 10 miRNA seed sequences within the 5,795 clusters.

Rank	miRNA	Seed motif	# clusters with seed match	Percentage of clusters with seed match	Percentage of background sequences with seed match	Signal/Noise
1	miR-30	TGTTTAC	162	2.79%	0.69%	4.04
2	miR-9	ACCAAAG	199	3.43%	0.88%	3.89
3	miR-181	TGAATGT	165	2.84%	1.03%	2.75
4	miR-26	TACTTGA	102	1.76%	0.57%	3.08
5	let-7	CTACCTC	225	3.88%	0.64%	6.06
6	miR-27	ACTGTGA	189	3.26%	0.98%	3.32
7	miR-708	AGCTCCT	46	0.79%	1.16%	0.68
8	miR-124	GTGCCTT	318	5.48%	0.7%	7.82
9	miR-34	CACTGCC	66	1.13%	0.90%	1.25
10	miR-125	CTCAGGG	85	1.46%	1.05%	1.39

### FET family protein PAR-CLIP datasets

We also applied the MiClip method to the PAR-CLIP dataset described in Hoell, *et al.* [3]. The FET family proteins (FUS, EWSR1 and TAF15) are abundant and highly conserved RBPs involved in cancer biology and other diseases. The binding targets of the FET family proteins were studied using PAR-CLIP, in which 4-thiouridine was used for cross-linking, and induced T->C mutations as the marker mutations for protein-RNA interaction. The study discovered that FUS protein binding to RNAs is likely to involve secondary structures, suggesting that structural motifs might play an important role in protein-RNA recognition. We used MiClip to analyze this dataset and compared the performance of MiClip with the *ad hoc*, *PARalyzer* and *wavClusteR* methods. We utilized the data provided in another study [31] as the validation dataset where the binding strength of the FUS and EWSR1 proteins to RNA transcript of every gene was measured experimentally using RNA-seq.

**Figure 4:**
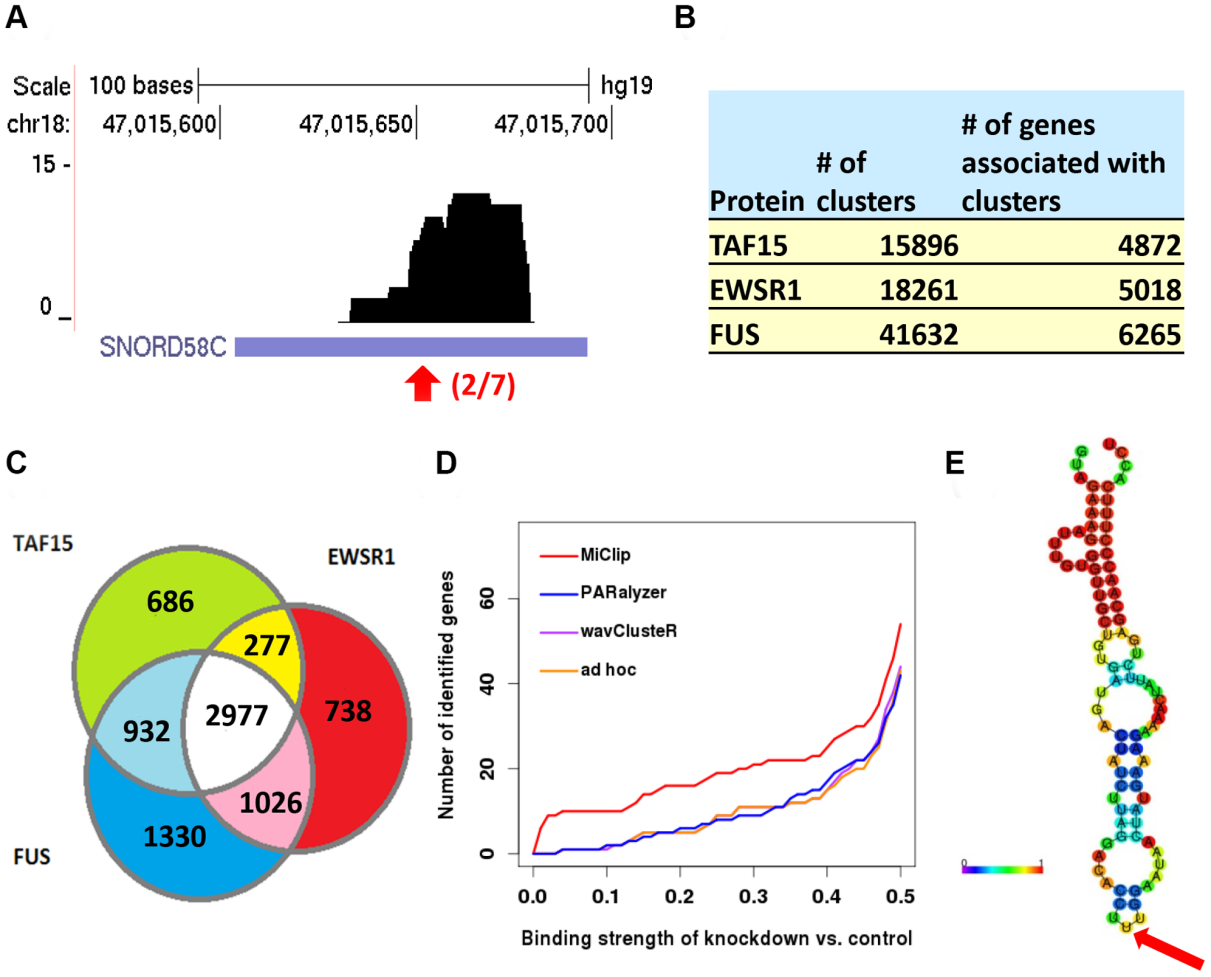
Results produced by MiClip on the FET family PAR-CLIP dataset. (a) Genome Browser visualization of an exemplary FUS cluster. Red arrow denotes the binding site. 2 out of 7 tags on this base have T->C mutations. (b) The number of reliable CLIP clusters identified in each sample and the number of genes whose gene bodies contain at least one cluster. (c) A Venn diagram of the overlap between gene targets identified by MiClip for TAF15, EWSR1 and FUS. (d) Number of target genes identified by *MiClip*, *PARalzyer*, *wavClusteR* and the *ad hoc* method in the FUS experiment. The x-axis is the cutoff ratio of the amount of RNA sequenced in the knockdown *vs.* control condition from the Han, *et al.* experiment. To be fair, genes are sorted first by the number of overlapping significant CLIP clusters, and ties are then sorted by the number of bases in each gene that have non-zero coverage of CLIP clusters. The top 5000 genes found by each tool were used for comparison. (e) The secondary structure of *SNORD58C* predicted by the RNAfold online server. The color bar shows the base-pair probabilities. The red arrow points to the identified binding site by MiClip.

The sequencing reads for the three proteins were aligned to the hg19 reference genome, resulting in 4,459,260, 3,886,283 and 4,296,458 mapped reads for TAF15, EWSR1 and FUS proteins, respectively. The library sizes of the three TAF15, EWSR1 and FUS experiments are smaller than those of typical CLIP-Seq studies, and saturation of sites at this sequencing depth is not reached according to their own calculation [3]. As a result, the identified reliable clusters are, on average, smaller in size compared to the AGO HITS-CLIP dataset, with an example of a FUS cluster given in **Fig. 4a**. Even so, MiClip still performed efficiently and accurately on these datasets. MiClip identified 15,896, 18,261 and 41,632 reliable clusters with binding sites for TAF15, EWSR1 and FUS, respectively. These three sets of clusters are associated with 4,872, 5,018 and 6,265 genes, respectively (**Fig. 4b**). The overlap of these target genes is significant (**Fig. 4c**, pv<1E-16, by hyper-geometric test). These results show that the binding patterns of the FET family proteins are similar to each other, which is reasonable and expected, because TAF15, EWSR1 and FUS have similar structures and highly conserved RNA recognition motifs.

Then we compared the performance of *MiClip* with *wavClusteR*, *PARalyzer* and the *ad hoc* method used in the original publication. *Piranha* [32] is another CLIP-Seq analysis software. However, the numbers of RBP binding sites identified by Piranha are too small (<1000 for all possible model options) at a P value cutoff of 0.05. Therefore, it is not included in this analysis. We compared the binding strengths of target genes identified from the PAR-CLIP datasets by the four methods using the external validation data from Han, *et al.* [31]. In the Han study, the authors used biotinylated isoxazole (b-isox) chemical to form RNA granule-like aggregates in cell lysates so that proteins, as well as bound RNAs, were precipitated upon exposure of lysates to the b-isox chemical. By sequencing these RNAs in the control and the knockdown conditions for a specific protein, the authors were able to investigate the binding strength of the RBP to all gene targets. In brief, the lower the ratio of the read count of an RNA transcript in knockdown *vs.* control conditions, the more likely the protein will bind to this RNA. We compared how many target genes identified by the four methods are differentially bound by applying a series of cutoff ratio values in the Han, *et al.* dataset. Genes with ratio smaller than a cutoff will be defined as true targets. We kept the top 5,000 genes found by the clusters identified by each tool for the FUS dataset. Then we applied a cutoff ratio, for example 0.4, to define truly bound genes, the 5,000 genes found by the *MiClip* approach contain 25 true binding targets under the cutoff value of 0.4, which is 67% more than the *ad hoc* method, 47% more than the *PARalyzer* method and 67% more than the *wavClusteR* method. By applying a series of cutoff ratios from 0–0.5 for differentially bound genes, we found that *MiClip* performed better than the *ad hoc*, *PARalyzer* and *wavClusteR* methods in identifying target genes of FUS and EWSR1 (**Fig. 4d** and **Fig. S3** in **File S1**). As an example of a reliable cluster identified by MiClip, but not by *wavClusteR*, *PARalyzer* or the *ad hoc* method, the base coverage of a CLIP cluster around *SNORD58C* is shown in **Fig. 4a**. The knockdown *vs.* control ratio for this gene is extremely small (equals 0.00016), and the predicted structure of *SNORD58C* by *RNAfold* shows that this snoRNA folds into a secondary structure where the identified binding site resides at the very tip of the structure (**Fig. 4e**). So, this gene is likely to be a true FUS binding target identified by MiClip, but it was missed by the other methods.

### FMR1 PAR-CLIP dataset

We extended the application of *MiClip* and comparison to other available softwares to another PAR-CLIP dataset, where the RBP of interest is FMR1 [33]. Loss of expression of FMR1 is associated with Fragile X syndrome [34]. FMR1 is one member of the FMR1 RBP family proteins including FMR1, FXR1, and FXR2. FMR1 encodes for multiple isoforms and the authors conducted PAR-CLIP experiments on isoform 1 and isoform 7 for both wild-type proteins and I304N mutant proteins. Through bioinformatics and experimental validation, the authors identified two major binding motifs of FMR1, ACTK (K = G or A) and WGGA (W = A or T). Morever, the authors found that ACTK and WGGA motifs are likely to occur in repeats and that ACTK and WGGA motifs often occur together with each other in short windows [33]. Here, we focus the application of MiClip on the isoform 7 wild-type protein experiment. We downloaded the sequencing files from GSE39686, trimmed the adapters and aligned the sequencing data to the hg19 genome using Bowtie [35]. This resulted in ∼5.5M mapped reads for following analysis.

**Figure 5:**
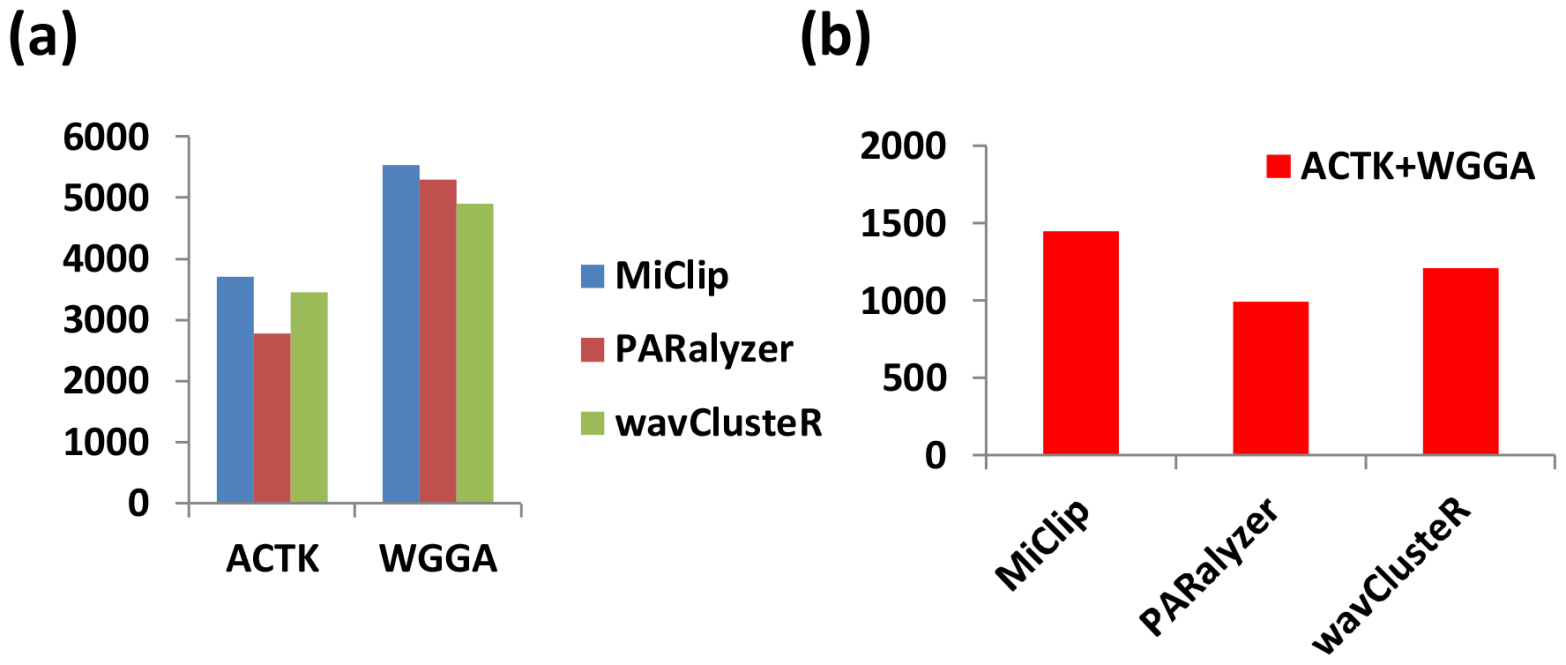
Comparison results of MiClip with PARalyzer and wavClusteR on the FMR1 PAR-CLIP dataset. (a) The total number of occurrences of ACTK motifs and WGGA motifs in the nucleotide sequences covered by the top 11,212 clusters identified by each software. One sequence may contain multiple matches and contribute more than one to the total count. (b) The total number of nucleotide sequences that contain at least one ACTK motif and at least one WGGA motif, out of all the 11,212 sequences for each software.

We ran *MiClip*, as well as *PARalyzer* and *wavClusteR*, on the alignment data. *MiClip* identified a total of 11,212 significant clusters with reliable binding sites. *PARalyzer* scored all 174,330 clusters with a self-defined value called ModeScore and *wavClusteR* identified a total of 69,830 significant clusters. For a fair comparison, we ranked the clusters identified by *PARalyzer* by ModeScore provided by the software and ranked the clusters identified by *wavClusteR* by total tag intensity over each CLIP cluster region. We picked the top 11,212 clusters identified by each software and shortened or extended each cluster towards its middle position to a uniform length of 30 bp. Then we scanned for the total number of occurrences of ACTK and WGGA motifs in the nucleotide sequences covered by these chosen clusters (one sequence might contain multiple motifs). **Fig. 5a** shows that the nucleotide sequences of clusters found by *MiClip* contain more ACTK motifs and WGGA motifs than those found by *PARalyzer* and *wavClusteR*. Furthermore, we calculated the number of sequences that contain both ACTK and WGGA motifs, out of all nucleotide sequences generated by each software. Fig. 5b shows that *MiClip* found more such sequences that contain both motifs than *PARalzyer* and *wavClusteR*. Again, these results suggest that *MiClip* performs better than the other two softwares.

## Discussion

In this study, we presented the MiClip approach for identifying reliable protein-RNA binding sites and clusters in CLIP-Seq datasets, including both HITS-CLIP and PAR-CLIP. MiClip is a model-based approach that can identify high-confidence binding sites using probability scores. Different from crosslinking-induced mutation sites (CIMS) analysis [8] that only looks at mutation rates, MiClip approach analyzes both tag counts and numbers of mutations simultaneously to improve detection power. It employs two HMMs, one for searching enriched regions at 5 bp resolution, and the other for searching binding sites at single base-pair resolution. The two-stage approach handles large sequencing data efficiently, while identifying binding sites at high-resolution. Our study shows that the MiClip approach performs better than the *wavClusteR*, *PARalyzer* and the *ad hoc* methods used in the original publications. One potential of MiClip is that it requires only 2 parameters to control the model fitting. The first one is the cutoff for truncating the counts of bins with extremely large count data (for the first HMM) and the second one is the parameter c in parameter estimation (for the second HMM). In comparison, *PARalyzer* requires more than 8 parameters, and *wavClusteR* requires 3 parameters to control model fitting. Some of these parameters are not intuitive and informative, so it could be difficult to decide the best values for these parameters. Usually the more parameters there are, the more difficult and confusing it will be. In the *MiClip* software, we provided default values for the 2 parameters, based on our experience with several CLIP-Seq datasets.

Choosing the characteristic marker mutation is important when analyzing CLIP-Seq datasets. The marker mutation type for PAR-CLIP dataset is easy to determine, because it depends solely upon the type of analog used in the experiment. However, for HITS-CLIP experiments, choosing the right type of mutation as the marker for binding events could be difficult. According to Zhang, *et al.* [8], deletion is the marker mutation for AGO and Nova HITS-CLIP experiments, but this may not hold true for other proteins. In fact, our unpublished data shows that for a certain human protein, we might need to include all types of mutations as marker mutations, because a well-defined target transcript bound by this protein contains large and comparable amounts of deletions, insertions and substitutions. In such cases, it is a good idea to run *MiClip* multiple times, each time specifying a different marker mutation or mutation combination, in order to compare the results and see which setting leads to the most reasonable results.

Compared with *MiClip*'s and *PARalyzer*'s underlying statistical models, *wavClusteR* has an additional feature for genomic positions with very high rates of T->C substitutions for modeling SNPs in the reference genome. The proportion of mutant tags with specific substitution, out of total tags on each base, is defined as relative substitution frequency (RSF) by *wavClusteR* method [16]. In the Mov10 dataset used in its original publication, the authors discovered that the number of genomic positions with T->C substitutions is similar to the number of genomic sites with other types of substitutions in the high RSF interval. Consequently, the authors included one component in the statistical model to represent this part, because they believed that these mutation sites with high rates of substitutions are questionable, and could be SNPs in the reference genome. However, the inherent probability of observing T->C mutation might be very different from the inherent probabilities of other kinds of substitutions, especially for cancer cell lines [36], making this approach problematic. Also, we found that in the EWSR1 dataset, the number of T->C mutations is still much larger than the number observed for any other type of substitutions in the [0.8–1] RSF interval (**Fig. S4a** and **Fig. S4b** in **File S1**). So, in our dataset, the majority of high RSF T->C mutation sites may still be true binding sites. This discrepancy could be caused mainly by the effect of different proteins and experimental designs in these two different studies. Importantly, the more convincing way to exclude false positives introduced by SNPs is to screen identified binding sites by known SNPs that exist in the experimental system according to other reliable information. For example, one can filter out SNP artifacts by conducting control experiments (e.g. RNA with no cross-linking) and align sequences to reference genome to uncover non-experimentally induced mutations. We provide one function *MiClip.snp* in the *MiClip* package to implement this functionality when such control experiment is available.

The MiClip algorithm takes full advantage of the unique properties of HMM, which is the core of the MiClip algorithm. First, HMM is a powerful method to identify hidden states with spatial dependencies between neighboring observations. CLIP clusters formed by overlapping short tags should have inherent spatial dependency features. In **Fig. S1a**, bins with tag intensity of 5 can be inferred either as enriched or non-enriched with similar probabilities. Bins with tag intensity of 5 should have higher probability of being inferred as enriched when their neighboring bins have bigger tag counts, which is how spatial dependency plays a role in statistical inference. For inference of enrichment, we implemented the Poisson model. For future studies, it would be interesting to investigate whether a Negative Binomial mixture model could help improve the inference accuracy. Secondly, protein binding events will lead to sequencing tag pile up, as well as sequencing mismatches in a random process, which can be naturally reflected as an emission function. In **Fig. S1b**, genomic sites with mutation ratio around 0.18 have similar probabilities of being inferred as binding sites or non-binding sites. Here, using binomial distributions to model the number of mutations, while considering the total tag counts, is better than looking at the mutation rate alone. As a simplified example, let us assume the mutation probabilities are 0.2 and 0.1 for a binding site and a non-binding site, respectively. The probability of observing 3 mutations from 10 tags is 0.201 and 0.057 at a binding and non-binding site, respectively, while the probability of observing 30 mutations from 100 tags is 0.0052 and 1.84e-8 at a binding and non-binding site, respectively. As a result, although the mutation rates are the same (equals 0.3), the probability of being a binding site for a site with 10 tag counts is different from a site with 100 tag counts. Overall, MiClip's analytic power is derived from the appropriate use of HMM. Besides, this model-based approach is able to provide a probability value for each identified binding site (**Fig. 2b**), which helps researchers plan subsequent experiments.

Following MiClip analysis, downstream analyses could provide new insights into binding sequence motifs or structural features, gene targets and enriched regulatory pathways, as well as many other aspects of RNA regulation for the protein of interest. Recently, discovering transcriptional modules by integrating binding information from ChIP-Seq or ChIP-chip experiments, and expression data from RNA-Seq or DNA microarray experiments, has gained a lot of attention [37], [38]. The inclusion of RNA-binding data from CLIP-Seq experiments could greatly facilitate modelling of gene regulatory networks. In other studies, CLIP-Seq data have been utilized to analyze RNA splicing events [39], [40] and miRNA targeting sites [41]. In conclusion, the analysis of RNA-protein binding events by MiClip could provide a much improved systematic understanding of gene regulation processes when integrated with analyses of other types of data.

We note that the CLIP-Seq technology is still in its infancy. Up to now, no proper control experiments to estimate background have been proposed to normalize for transcript abundance. This is one of the long-standing problems of CLIP-Seq experiments. This problem is not addressed by the CLIP-Seq analysis tools available so far. As we don't assume that the control data are available, *MiClip* does not require a control input for analysis, either. However, one advantage of *MiClip* over other existing tools is that its second round of HMM employs binomial distribution of mutant and total tag counts to identify high-confidence binding regions. This partially avoids the problem of no control since the probability of mutation occurrences in the CLIP-Seq condition is independent of the transcript abundance inferred from the control condition but still carries information for identification of RBP binding sites.

RNA structures could potentially influence RBP-RNA binding events. However, not every RBP has well-defined RNA binding motif. Also the RNA binding motif could be vastly different for different RBPs. This prevents us from incorporating RNA structure motifs in our MiClip algorithm in a general manner. We suggest biologists and bioinformatics to look for patterns of RNA structure as well as RNA recognition motif in the significant CLIP clusters found by MiClip, or any other CLIP-Seq peak calling software. We will try to incorporate RNA structure motifs in the statistical modelling in future versions of MiClip.

The *MiClip* R package is designed to be flexible in analyzing both HITS-CLIP and PAR-CLIP datasets. In MiClip, the marker mutations introduced in HITS-CLIP and PAR-CLIP experiments could be defined as deletion, insertion, substitution, or some combination of these mutation types. For example, if the characteristic mutations induced for a specific protein are both deletions and insertions, the MiClip method can take both types of mutations into consideration at the same time. We also added an option in the *MiClip* package to incorporate background sequencing data for normalization purpose, if such data are available. Conducting background profiling of gene expression as a control for CLIP-Seq experiments has not become a standard procedure yet, but could be very helpful for improving the accuracy of the identification of RBP targeting sties. The package can handle both single-end and paired-end CLIP-seq data. Users could run this package on UNIX, Mac OS or PC machines. In addition, the *MiClip* package is highly efficient. It takes 45 minutes for *MiClip* to analyze the FUS PAR-CLIP dataset with sequencing depth ∼4.2 million reads, compared with *wavClusteR* which takes 2 hours to analyze the same dataset. For the AGO HITS-CLIP dataset with ∼26 million reads, *MiClip* only takes 60 minutes to process. Although it takes *PARalyzer* about 30 minutes to process the FUS dataset, it can only accept Bowtie-format output files from the Bowtie aligner, which severely limits its application.

Taken together, the MiClip approach provides a general, efficient and accurate method for identifying high confidence RBP binding sites and CLIP clusters in various versions of CLIP-Seq experiments. Moreover, the MiClip approach could be easily adapted for other substitution-based high-throughput sequencing datasets.

### Implementation

We have implemented the algorithm in an R package, *MiClip*. Part of the package is written in Perl to improve the efficiency and flexibility in handling large sequencing data. The *MiClip* package provides a function (*MiClip.snp*) to filter out the false positives caused by SNP artifacts if a control sample is supplied. Also, a helper R function to trim adaptor sequence from the 3′ end of Fastq format sequencing files is provided in the *MiClip* package. The package source, user manual and vignette have been documented on CRAN.

Running *MiClip* locally could avoid the trouble of uploading huge data to web-based servers for analysis. However, to help biologists with limited computation resources, we also developed a user-friendly web-based interface to MiClip (**Fig. S5** in **File S1**). This user-friendly web server is able to take BAM files, as well as SAM files, as input. The users do not need to download or install any software, and do not need to learn any programming skills to run the algorithm. This software is based on Galaxy platform [42]–[44], and all the analysis parameters will be automatically saved to ensure the reproducibility of the data analysis.

The manuals for both the R *MiClip* package and the Galaxy *MiClip* software are provided in **Text S1–S2 in File S1**.

## Supporting Information

File S1 **Supplementary data includes: Figures S1–S5, Table S1 and Text S1–S2.** **Table S1.** The number of predicted binding sites per cluster for all CLIP clusters identified to have at least one reliable binding site in the AGO HITS-CLIP dataset. **Figure S1.** Distribution of tag counts and mutation ratios in each state. **Figure S2.** Tag pileup of a “flat” cluster from the AGO HITS-CLIP dataset. **Figure S3.** Target genes identified by *MiClip*, *PARalyzer*, *wavClusteR* and the *ad hoc* method in the EWSR1 experiment. **Figure S4.** Numbers of mutant genomic sites with the specified substitutions and in the two RSF intervals. **Figure S5.** The workflow of the MiClip Galaxy server.
(PDF)
