# Supplementary material for: A Model-Based Approach to Identify Binding Sites in CLIP-Seq Data
Source: PLoS One. 2014 Apr 8;9(4):e93248. doi: 10.1371/journal.pone.0093248 (PMC3979666; doi:10.1371/journal.pone.0093248)
Supplement: File S1 — Supplementary data includes: Figures S1–S5, Table S1 and Text S1–S2. Table S1. The number of predicted binding sites per cluster for all CLIP clusters identified to have at least one reliable binding site in the AGO HITS-CLIP dataset. Figure S1. Distribution of tag counts and mutation ratios in each state. Figure S2. Tag pileup of a “flat” cluster from the AGO HITS-CLIP dataset. Figure S3. Target genes identified by MiClip, PARalyzer, wavClusteR and the ad hoc method in the EWSR1 experiment. Figure S4. Numbers of mutant genomic sites with the specified substitutions and in the two RSF intervals. Figure S5. The workflow of the MiClip Galaxy server. (PDF) [file pone.0093248.s001.pdf]

A Model-based Approach to Identify Binding Sites in CLIP-Seq Data

Tao Wang[1], Beibei Chen[1, 2], MinSoo Kim[1, 2], Yang Xie[1, 2] and Guanghua Xiao[1,*]

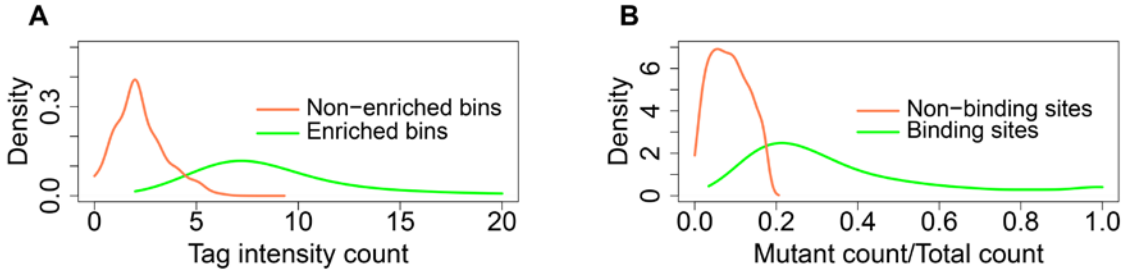

Sup Fig. 1. | Distribution of tag counts and mutation ratios in each state. (a) The distribution of tag intensity counts of enriched vs. non-enriched bins inferred by the first HMM. (b) The distribution of mutant rates of binding sites vs. non-binding sites inferred by the second HMM. For the sake of presentation, the 0s in the zero-inflated binomial part are not shown.

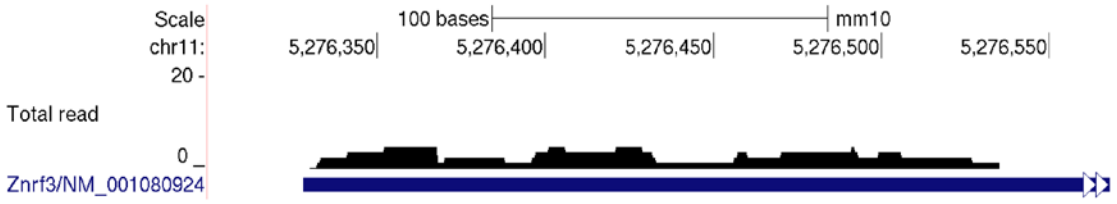

Sup Fig. 2. | Tag pileup of a "flat" cluster from the AGO HITS-CLIP dataset.

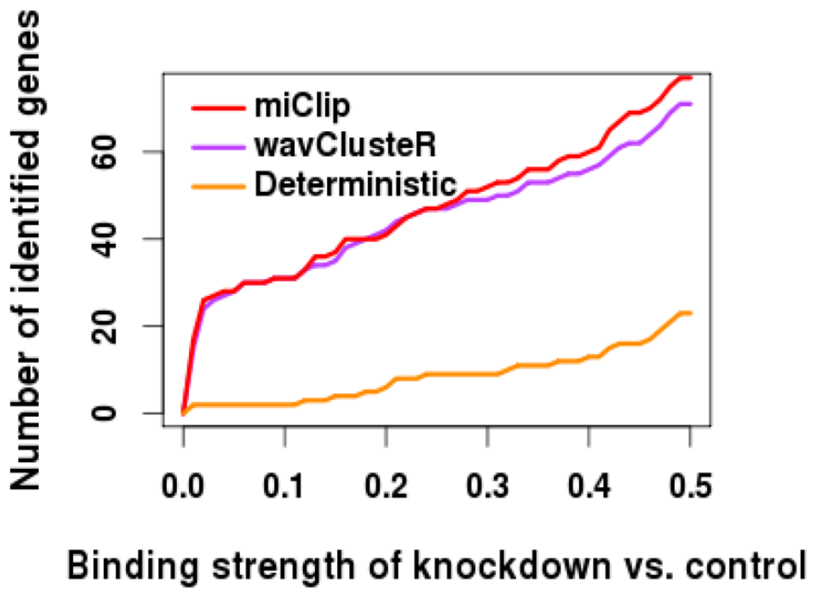

Sup Fig. 3. | Target genes identified by *MiClip*, *PARalyzer*, *wavClusteR* and the *ad hoc* method in the EWSR1 experiment. The x-axis is the cutoff ratio of the amount of RNA sequenced in the knockdown vs. control condition from the Han, et al experiment. Genes are sorted in the same way as in **Fig. 4d**. The top 3,500 genes found by each tool were used for comparison.

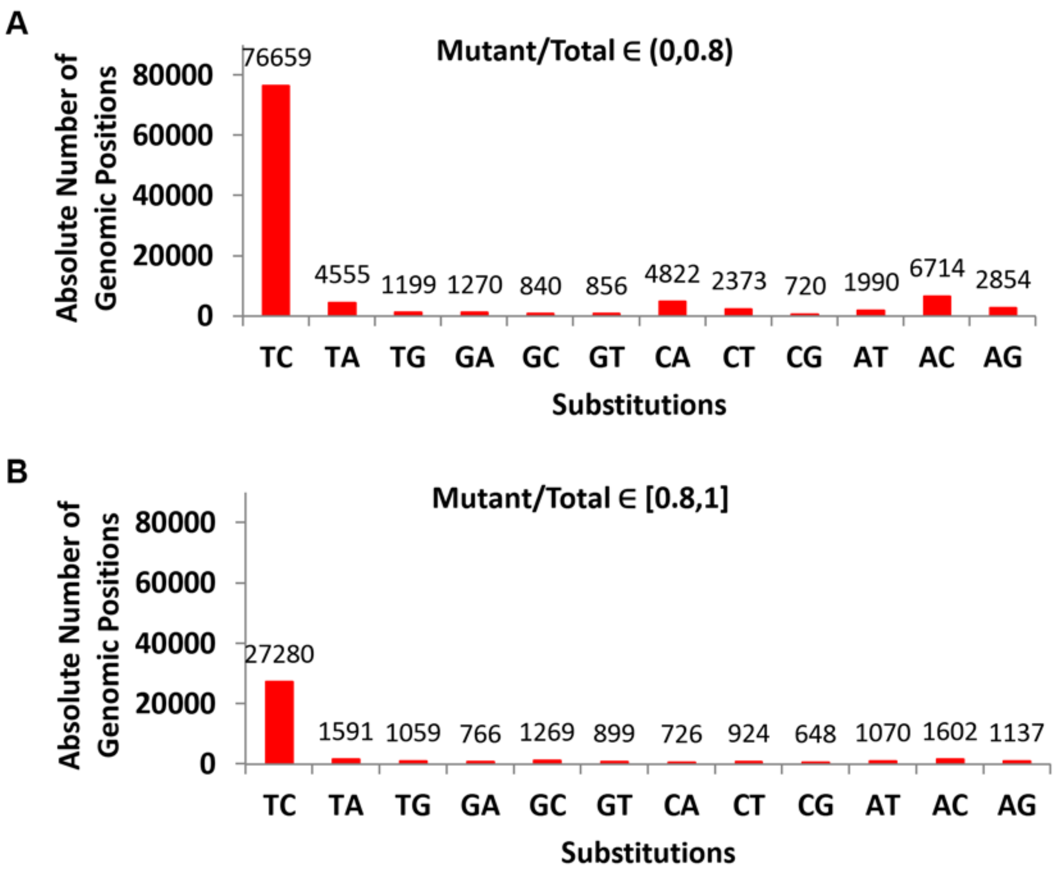

Sup Fig. 4. | Numbers of mutant genomic sites with the specified substitutions and in the two RSF intervals. The absolute numbers of genomic sites are shown on top of each bar.

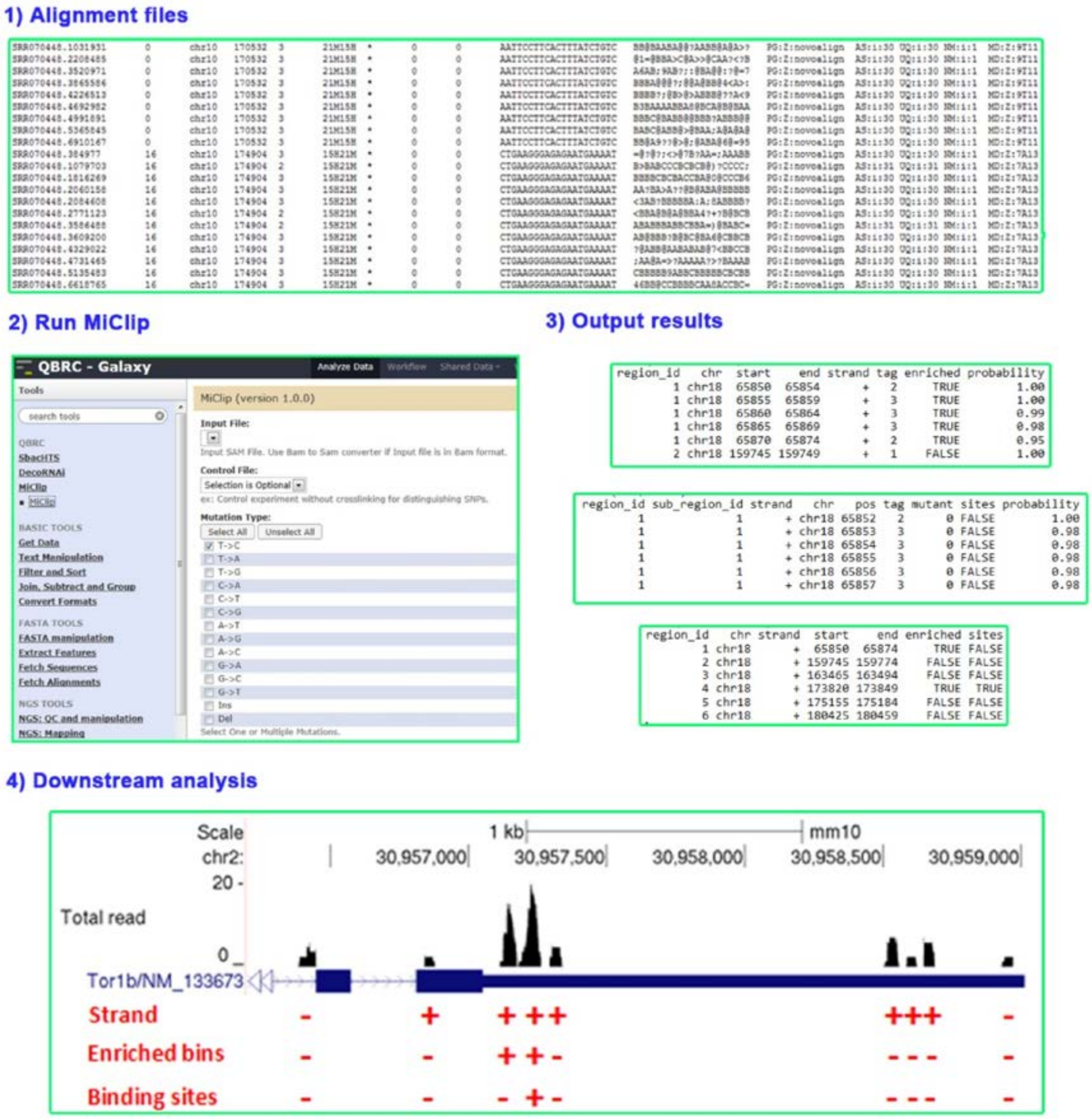

Sup Fig. 5. | The workflow of the MiClip Galaxy server. Raw sequencing file can be in SAM or BAM format. Users can analyze these CLIP-Seq files using the MiClip program implemented on the QBRC Galaxy server. The output consists of 3 or 4 files, depending on whether a control sequencing file is provided. Following the MiClip analysis, users can use their own scripts or other software to conduct downstream analysis.

Sup Table 1. | The number of predicted binding sites per cluster for all CLIP clusters identified to have at least one reliable binding site in the AGO HITS-CLIP dataset.

# predicted binding sites per cluster	# clusters
1	5001
2	648
3	103
>=4	43

The MiClip Manual for Galaxy

Tao Wang

March 29, 2013

Abstract

There has been increasing interest in the role of RNA-binding proteins in biological processes. Crosslinking and immunoprecipitation (CLIP) experiments have made it possible to identify binding sites of RNA-binding proteins in various cell culture and tissue types. The two most commonly used types of CLIP-Seq experiments are HITS-CLIP and PAR-CLIP. Here we present MiClip, an R package implemented on Galaxy server, for identification of binding sites in CLIP-Seq experiments. The MiClip package employs two rounds of Hidden Markov Model (HMM) to identify enriched regions and further high-confidence binding sites from raw sequencing data.

1 Alignment

Trim adaptors During CLIP-Seq experiments, RNAs are usually digested to short fragments. It is quite often for sequenced reads to have adaptor contamination at the 3' end, while it is relatively rare for the 5' end of short reads to have adaptor contamination. Thus, it is necessary to trim contaminating adaptor sequences from 3' end before running alignment. Users can use public softwares like Trimmomatic to trim adaptors.

MD fields The raw sequencing file can be single-end or paired-end in basespace or colorspace. Any mainstream alignment software can be used to align the short reads. The output format must be SAM/BAM format and in basespace. MiClip can work on both single-end and paired-end alignment files. The MiClip algorithm collects mutation information from the CIGAR and MD fields of each short read. The MD field is a string for mismatching positions characterized by MD:Z: towards the end of each entry in the alignment file. Please make sure the MD fields are present in the aligned reads. If not, the user should install and use samtools to populate the MD fields. However, this command runs very slowly. So it will be much better if the user can choose an alignment software which will give MD fields to all mapped tags in the very beginning. I myself only know that bowtie and novoalign produce correct MD fields and tophat cannot. It wont be a bad idea to try your aligner first on a small test dataset and see if MD field is attached before aligning all your samples.

Multiple mapping reads Multiple mapping reads are reads that can be mapped to more than one place in the genome. In the alignment process, the user can specify whether/how many hits per read to report in the alignment file while MiClip will take in all reported hits.

Mapping across splice junctions Reads that are mapped across splice junctions are discarded (these are different from reads that are mapped with short deletions). These reads typically only occupy less than 3% of total mapped reads. If you insist on analyzing these reads too, please map your reads to trancriptome and then analyze them using MiClip.

2 Input files

Input format The input file for MiClip is the SAM format file. However, BAM file, the binary version of SAM file, is compressed and much smaller than the SAM file. So we advise users to upload BAM files to Galaxy and convert BAM files to SAM files using the SAMtools (example shown later). Direct uploading of SAM files is also allowed but not recommended.
Paired-end mode For paired-end reads, the users must look at the sequencing files and provide the suffix for the forward strand and the backward strand. For example, the mate in the sequencing dataset may be named like ”694_122_1972-F3” and ”694_122_1972-F5-RNA”, where ”694_122_1972” is the id number of the mate, ”F3” means forward strand and ”F5-RNA” means backward strand. Then the suffix should be ”F3,F5-RNA” or ”F3,F5-RNA” or ”3,5-RNA”. Sometimes, the aligner will trim the suffix. For example, ”HWI-ST188:8:2217:5190:132924#0/1” and ”HWI-ST188:8:2217:5190:132924#0/2” are one mate and certain aligners will only write ”HWI-ST188:8:2207:5196:132924#0” for both segments in the alignment file. In such cases, please set the suffix to ”0,0” or ”#0,#0”. The point is to make the remaining part of the read names the same for a mate.
SNPs The analysis of the mutation-based CLIP-Seq datasets could be obscured by SNPs in the tissue or cell line. We provide an option for users to upload the alignment data of a control experiments (e.g. RNA with no cross-linking) and MiClip will mark those high confidence CLIP bindings sites which might actually be SNPs. Users can add additional quality control steps before alignment of the control data to the reference genome. MiClip will take the alignment file of the control condition and look for the same mutations as in the treatment sample. A null hypethesis is tested on each mutant site by MiClip in order to extract possible SNPs. Then the binding sites inferred by MiClip will be screened for these possible SNPs (mutant sites that are not inferred as binding sites are ignored). A column will be added to the ”sites.csv” file in the final output specifying whether a binding site could actually be a SNP. And another column will be added to ”clusters.csv” in the final output specifying those clusters, at least one of whose binding sites could be a SNP. Another ”snp.csv” file will also be attached that contains information of all the possible SNP sites extracted from the control experiment file.

3 Parameters

Below are the explanations of all the input parameters.
Input File The input file. This file must be in SAM format and basespace but either single-end or paired-end mode.
Control File The alignment file of the control experiment (if available).
Mutation Type The marker mutation for the CLIP-Seq experiment. It can be any one and combination of the 12 types of substitutions plus deletion or insertion. ”T-¿C” denotes T-to-C substitution, ”Ins” denotes insertion of any length and ”Del” denotes deletion of any length. The default is ”T2C”.
Sequence is Pair-End Whether the sequencing data is paired-end. Default is FALSE.
Suffix of Paired-End Read The suffix of the paired-end read data. For example, if the mate pairs in the SAM file are named as ”1_2_100708_26_788_F3”, ”1_2_100708_26_788_F5-RNA”, etc, suffix can either be ”F3,F5-RNA” or ”_F3,_F5-RNA”.

Bin Step Size In the first HMM, all clusters will be divided into bins of the same length of step bp and HMM will work to distinguish enriched bins from non-enriched ones. Default is 5 and for larger dataset(¿20M mapped reads) it is better to set step to a value between 10-15.
Empirical Used to help model fitting in the first HMM. Default is "auto" which lets the algorithm decides its value. It can be set to the estimated minimal number of overlapping tags for a reliable enriched CLIP cluster if default does not work. A higher value will lead to more conservative estimation.
Mixture Model Cutoff The cutoff for fitting the mixture model in the second HMM. It can be set to the estimated minimal proportion of mutation tags vs. total tags for a binding site to be reliable. Larger values will lead to more conservative predictions. It should be between 0 and 1 and the default is 0.2.
Max Number of Tag Count The maximum number of tag counts in a bin or on a base. This is used to keep calculation within the dynamic range of R. If this number is too large, probability values which are very small will become zero. Default is 100.
Max Number of HMM Iterations The maximum number of iterations allowed for both HMM iterations. Default is 20.
Convergence Cutoff The cutoff for reaching convergence. Default is 0.01.

4 Output format

MiClip will give 3 or 4 csv files as output depending on whether a control alignment file is provided.
enriched The output of the first HMM. "region_id" is the id number generated for each cluster. "chr", "strand", "start" and "end" specify the genomic location of each bin. "tag" is the rounded average tag count in each bin. "enriched" and "probability" are the inference results.
sites The output of the second HMM. "region_id" is the id number generated for the cluster where each base resides. "sub_region_id" is the id number of the concatenated segment within enriched clusters. "chr", "strand" and "pos" specify the genomic location of each base. "tag" is the read count on each base and "mutant" is the mutant count on each base. "sites" and "probability" are the inference results.
clusters The summary of results for all CLIP clusters. clusters contains information of chromosome, strand, start position, end position, whether or not contains enriched bins and whether or not contains binding sites.
snps This file will be generated if control alignment file is provided. It contains information of all the possible SNP sites extracted from the control experiment file.

5 Demo

The red rectangle in Fig. 1 shows the MiClip software implemented on the QBRC Galaxy server.

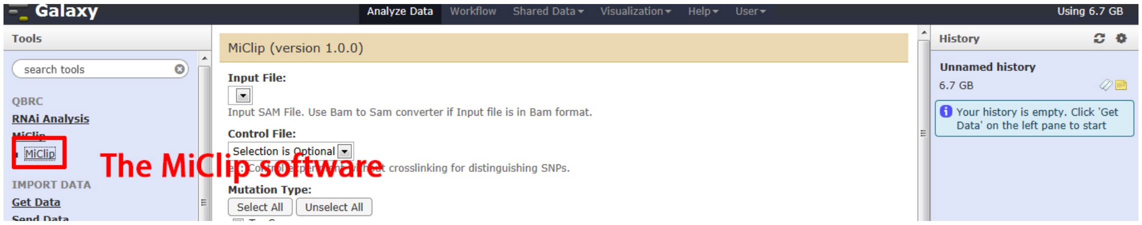

Figure 1: The MiClip software on Galaxy

The red rectangle in Fig. 2 shows the "Shared Data" where a small demo dataset is stored. Please click on it and go to "Data Libraries".

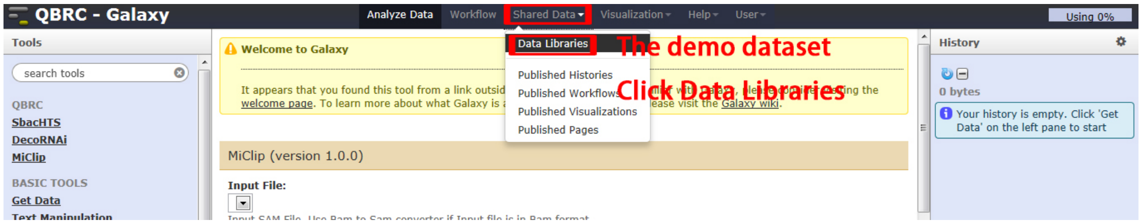

Figure 2: Import the demo dataset

In the "Data Libraries" shown as in Fig. 3, Select MiClip Demo Data.

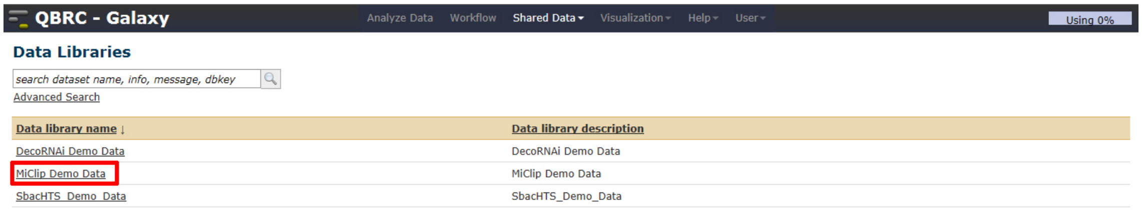

Figure 3: Import the demo dataset

In the "Data Library MiClip" shown as in Fig. 4, check both datasets and click "Go". Then go back to MiClip.

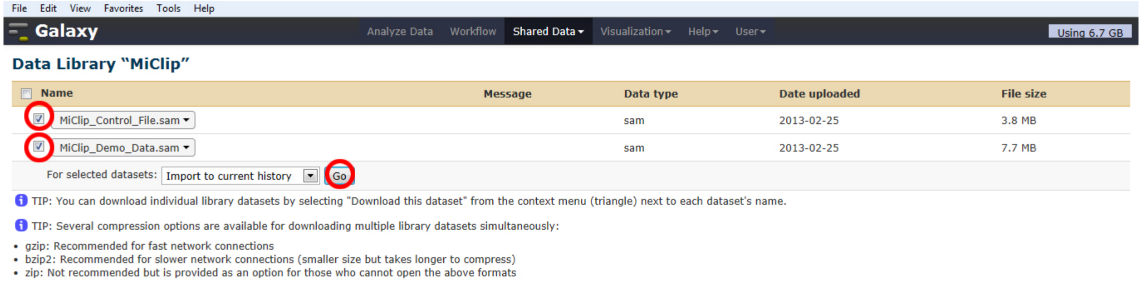

Figure 4: Import the demo dataset

Go back to MiClip

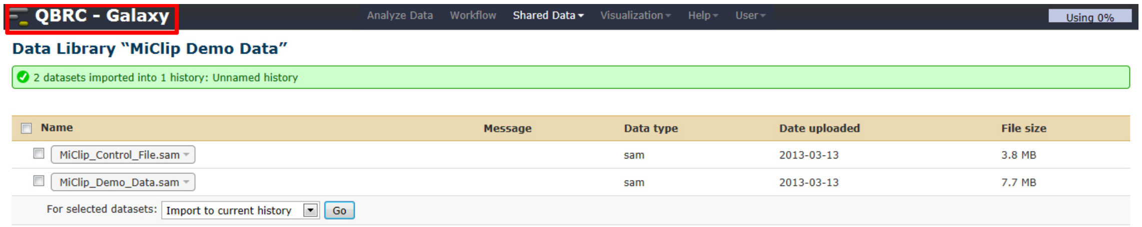

Figure 5: Import the demo dataset

When uploading the user's own dataset, please use "Get Data" to upload as in Fig. 6. BAM files (recommended) or SAM files can be uploaded in "Get Data". And BAM files can be converted to SAM files by using "NGS: SAM Tools".

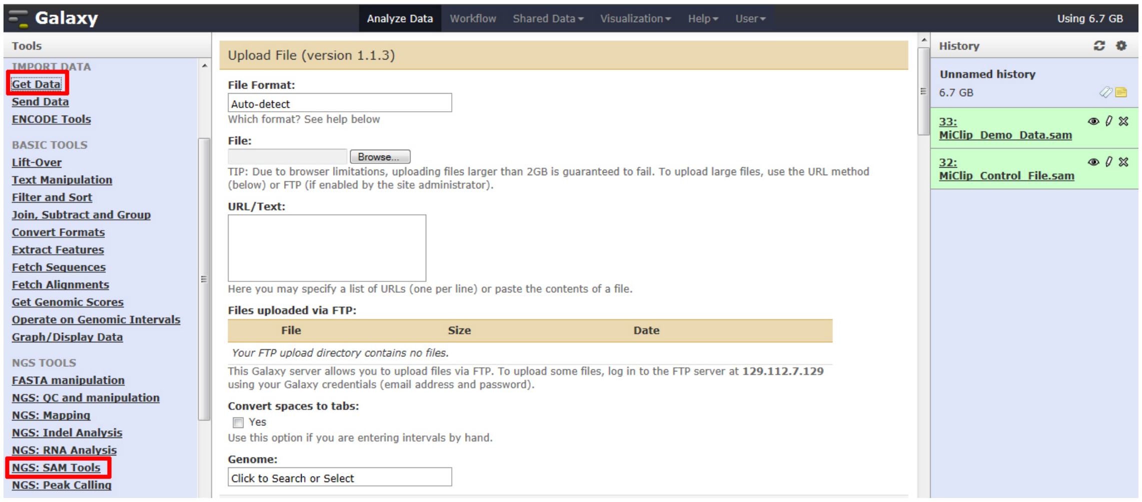

Figure 6: Upload user's own data

To run MiClip on the demo dataset, open MiClip first. Set "Input File" to "MiClip_Demo_Data.sam", set "Control File" to "MiClip_Control_Data.sam" and set the "Mutation Type" to "Del" and then click "Execute". All the other parameters will remain default for this demo case. This is shown in Fig. 7.

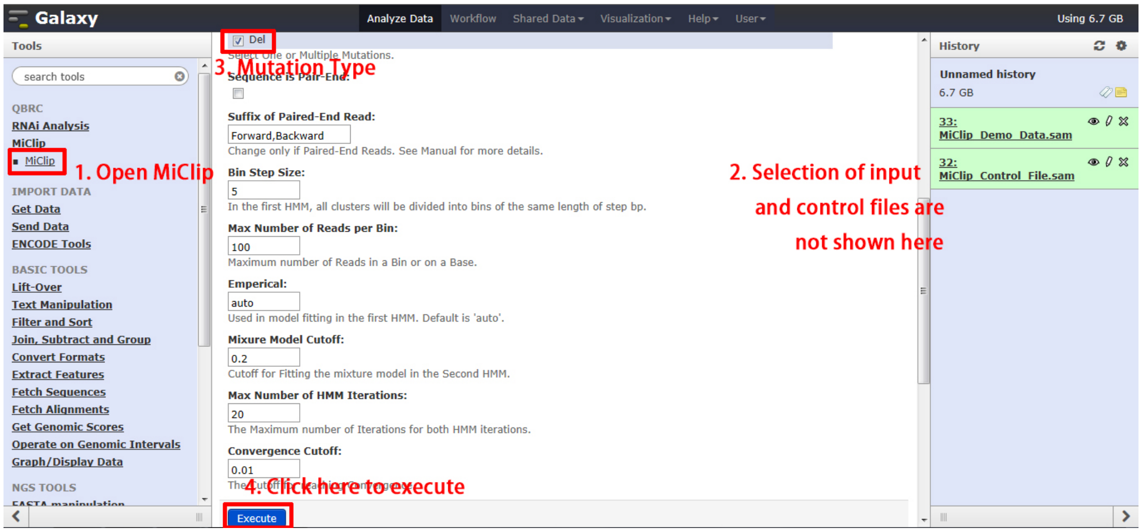

Figure 7: Run MiClip

The SAM file will be submitted to MiClip and when the analysis is done, the result will be shown in the upper right corner of the screen (Fig. 8). If the analysis is successful, this block should be green and if there is any error incurred, the block will be red with error messages printed.

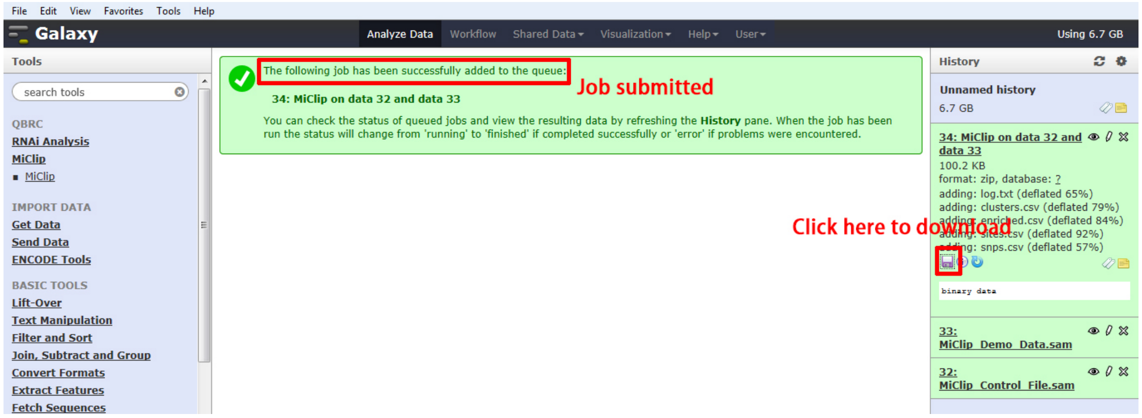

Figure 8: Run MiClip

The output files. Refer to Section 4 on the output format.

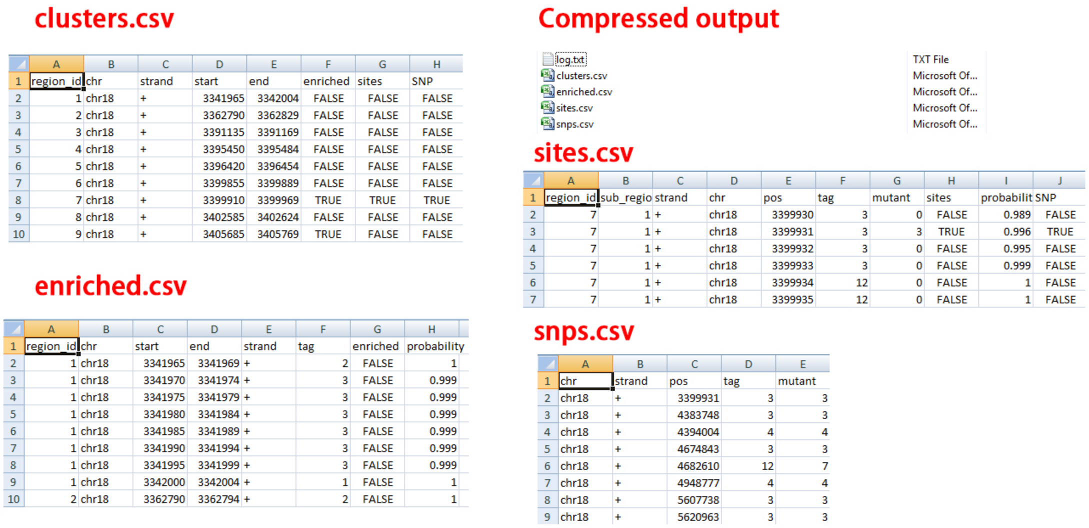

clusters.csv

region_id	chr	strand	start	end	enriched	sites	SNP
1	chr18	+	3341965	3342004	FALSE	FALSE	FALSE
2	chr18	+	3362790	3362829	FALSE	FALSE	FALSE
3	chr18	+	3391135	3391169	FALSE	FALSE	FALSE
4	chr18	+	3395450	3395484	FALSE	FALSE	FALSE
5	chr18	+	3396420	3396454	FALSE	FALSE	FALSE
6	chr18	+	3399855	3399889	FALSE	FALSE	FALSE
7	chr18	+	3399910	3399969	TRUE	TRUE	TRUE
8	chr18	+	3402585	3402624	FALSE	FALSE	FALSE
9	chr18	+	3405685	3405769	TRUE	FALSE	FALSE

enriched.csv

region_id	chr	start	end	strand	tag	enriched	probability
1	chr18	3341965	3341969	+	2	FALSE	1
1	chr18	3341970	3341974	+	3	FALSE	0.999
1	chr18	3341975	3341979	+	3	FALSE	0.999
1	chr18	3341980	3341984	+	3	FALSE	0.999
1	chr18	3341985	3341989	+	3	FALSE	0.999
1	chr18	3341990	3341994	+	3	FALSE	0.999
1	chr18	3341995	3341999	+	3	FALSE	0.999
1	chr18	3342000	3342004	+	1	FALSE	1
2	chr18	3362790	3362794	+	2	FALSE	1

Compressed output

log.txt	TXT File
clusters.csv	Microsoft Of...
enriched.csv	Microsoft Of...
sites.csv	Microsoft Of...
snps.csv	Microsoft Of...

sites.csv

region_id	sub_regio	strand	chr	pos	tag	mutant	sites	probabilit	SNP
7	1	+	chr18	3399930	3	0	FALSE	0.989	FALSE
7	1	+	chr18	3399931	3	3	TRUE	0.996	TRUE
7	1	+	chr18	3399932	3	0	FALSE	0.995	FALSE
7	1	+	chr18	3399933	3	0	FALSE	0.999	FALSE
7	1	+	chr18	3399934	12	0	FALSE	1	FALSE
7	1	+	chr18	3399935	12	0	FALSE	1	FALSE

snps.csv

chr	strand	pos	tag	mutant
chr18	+	3399931	3	3
chr18	+	4383748	3	3
chr18	+	4394004	4	4
chr18	+	4674843	3	3
chr18	+	4682610	12	7
chr18	+	4948777	4	4
chr18	+	5607738	3	3
chr18	+	5620963	3	3

Figure 9: Output format

The MiClip package

Tao Wang
The University of Texas Southwestern Medical Center,
5323 Harry Hines Boulevard Dallas, Texas, 75390
tao.wang@utsouthwestern.edu

March 28, 2013

Abstract

There has been increasing interest in the role of RNA-binding proteins in biological processes. Crosslinking and immunoprecipitation (CLIP) experiments have made it possible to identify binding sites of RNA-binding proteins in various cell culture and tissue types. The two most commonly used types of CLIP-Seq experiments are HITS-CLIP and PAR-CLIP. Here we present MiClip, an R package for identification of binding sites in CLIP-Seq experiments. The MiClip package employs two rounds of Hidden Markov Model (HMM) to identify enriched regions and further high-confidence binding sites from raw sequencing data.

Contents

1 Installation **2**

2 Preparation of input files **2**
2.1 Trimming adaptor . . . 2
2.2 Alignment . . . 2
2.3 Multiple-mapping reads . . . 3
2.4 Mapping across splice junctions . . . 3
2.5 Paired-end reads . . . 3

3 Running *MiClip* **3**
3.1 Construct a *MiClip* class object for following analysis . . . 3
3.2 Read raw sequencing data and mutation data . . . 4
3.3 Identify enriched bins . . . 4
3.4 Identify binding sites . . . 4
3.5 Screening for SNPs . . . 5

4 Output of *MiClip* **5**
4.1 Output format . . . 5
4.2 SNPs . . . 7
4.3 `MiClip.sum` . . . 8

5 Session Info **8**

1 Installation

R (`http://www.r-project.org/`) needs to be installed first for *MiClip* and the installation of the *MiClip* package follows the regular method for R package installation.

However, *MiClip* also requires Perl to be installed. Perl should ship along with any standard UNIX and MacOS distribution. But Windows users probably need to install Perl themselves (`http://www.perl.com/`). The users can type the following line in the command console to check if Perl has been installed properly.

```
perl -v
```

2 Preparation of input files

2.1 Trimming adaptor

During CLIP-Seq experiments, RNAs are usually digested to short fragments. It is quite often for sequenced reads to have adaptor contamination at the 3' end, while it is relatively rare for the 5' end of short reads to have adaptor contamination. Thus, it is necessary to trim contaminating adaptor sequences from 3' end before running alignment. Users can use published softwares like Trimmomatic [3] to trim adaptors.

We encourage users to use these more professional softwares, but we also provide a very simple helper function to remove adaptor sequence. Here we use a small portion of the data from [1] for demonstration. A new file with ".removed" suffix will be generated in the same folder as the original file. On my computer, it takes 40 minutes to process a fastq file of 20 million reads (80 million lines).

```
> library("MiClip")
> MiClip.adaptor(file=system.file("extdata/test.fastq",package="MiClip"),
+                adaptor="TGGAATTCTCGGGTGCCAAGGAACTCCAGTCAC")
```

2.2 Alignment

The raw sequencing file can be single-end or paired-end in basespace or colorspace. Any mainstream alignment software can be used to align the short reads. The output format must be SAM/BAM format and in basespace. *MiClip* can work on both single-end and paired-end alignment files. In the case where the user wishes to pool the alignment files from several experiments, the user can just concatenate the SAM files simply by typing the following in the command console.

```
cat example1.sam example2.sam > example.sam
```

The MiClip algorithm collects mutation information from the CIGAR and MD fields of each short read. The MD field is a string for mismatching positions characterized by "MD:Z:" towards the end of each entry in the alignment file (`http://samtools.sourceforge.net/SAM1.pdf`). Please make sure the MD fields are present in the aligned reads. If not, the user should install and use samtools to populate the MD fields, please see the instructions by typing the following command in command console.

```
samtools fillmd
```

However, this command runs very slowly. So it will be much better if the user can choose an alignment software which will give MD fields to all mapped tags in the very beginning. I myself only know that bowtie and novoalign produce correct MD fields and tophat cannot. It won't be a bad idea to try your aligner first on a small test dataset and see if MD field is attached before aligning all your samples.

2.3 Multiple-mapping reads

"Multiple mapping" reads are reads that can be mapped to more than one place in the genome. In the alignment process, the user can specify whether/how many hits per read to report in the alignment file while MiClip will take in all reported hits.

2.4 Mapping across splice junctions

Reads that are mapped across splice junctions are discarded (these are different from reads that are mapped with short deletions). These reads typically only occupy less than 3% of total mapped reads. If you insist on analyzing these reads too, please map your reads to trancriptome and then analyze them using *MiClip*.

2.5 Paired-end reads

For paired-end reads, the users must look at the sequencing files and provide the suffix for the forward strand and the backward strand. For example, the mate in the sequencing dataset may be named like "694_122_1972-F3" and "694_122_1972-F5-RNA", where "694_122_1972" is the id number of the mate, "F3" means forward strand and "F5-RNA" means backward strand. Then the suffix should be "F3" and "F5-RNA" or "F3" and "F5-RNA" or "3" and "5-RNA".

Sometimes, the aligner will trim the suffix. For example, "HWI-ST188:8:2217:5190:132924#0/1" and "HWI-ST188:8:2217:5190:132924#0/2" are one mate and certain aligners will only write "HWI-ST188:8:2207:5196:132923#0" for both segments in the alignment file. In such cases, please set the suffix to "" and "" or "#0" and "#0". The point is to make the remaining part of the read names the same for a mate.

3 Running *MiClip*

3.1 Construct a *MiClip* class object for following analysis

The analysis of *MiClip* starts by constructing a `MiClip` object. Here we used a small portion of the single-end HITS-CLIP data provided in [2] for demonstration purpose.

```
> library("MiClip")
> test=MiClip(file=system.file("extdata/test.sam",package="MiClip"),mut.type="Del")
>
> # for paired-end data
> # test=MiClip(file="test.sam",paired=TRUE,suffix=c("F3","F5-RNA"))
```

This command returns a `MiClip` object for further analysis. Following sections will explain some of the available parameters in constructing this object. For detailed descriptions of all parameters, please refer to the *MiClip* manual.

One thing to note is that if you need to include the path name in the file name, the path name cannot start with anything like "∼". Namely, you must write the path name in full like "/home/project/test.sam" rather than "∼/project/test.sam".

3.2 Read raw sequencing data and mutation data

The `MiClip.read` function calls some embeded perl scripts to form clusters (CLIP clusters) by overlapping reads and collect tag pile-up as well as mutation information from the input SAM file. This process will usually take a few minutes depending on the size of the file.

```
> test=MiClip.read(test) # read raw data

Identifying clusters finished!
Generating bin file finished!
```

3.3 Identify enriched bins

The `MiClip.enriched` function first collects tag pile-up information on a `step` bp basis (bins) and estimates the paramters for a two-poisson mixture model for the count values. Because we are running a truncated part of the real data for demonstration, so the model estimation will not be accurate. Then the first Hidden Markov Model will try to identify the enriched bins vs. non-enriched bins in CLIP clusters.

```
> test=MiClip.enriched(test,quiet=FALSE) # identify enriched regions

Initialization of the first HMM finished!
>>>>>
Iterations of the first HMM finished!
Viterbi algorithm of the first HMM finished!
```

The `empirical` parameter is devised to adjust model estimation in this step. The default for `empirical` is "auto", which lets the algorithm decides its value. User can set this value to roughly the minimal number of overlapping tags for a "true" cluster according to user's experience and experimental design. Larger value will lead to more conservative predictions.

3.4 Identify binding sites

The `MiClip.binding` function first concatenates neighboring enriched bins and then expands each chain of adjacent bins into single base pairs. Then `MiClip.binding` collects the tag pile-up and mutant pile-up information on each base for estimation of a mixture model of one zero-inflated binomial distribution and a binomial distribution. Then the second Hidden Markov Model is run to identify significant binding sites.

```
> test=MiClip.binding(test,quiet=FALSE) # identify binding sites

Initialization of the second HMM finished!
>>>
Iterations of the second HMM finished!
Viterbi algorithm of the second HMM finished!
```

The `model.cut` parameter is devised to adjust model estimation in this step. The default for `model.cut` is 0.2. User can set this value to roughly the minimal proportion of mutant tag vs. total tag on true binding sites according to user's experience and experimental design. Larger value will lead to more conservative predictions.

3.5 Screening for SNPs

The *MiClip* package builds in a function `MiClip.snp` for distinguishing true binding sites from possible SNPs. The control can be a sequenced sample that is not processed by the crosslinking step. Optionally, users can do quality screening on the fastq sequencing file before alignment to the reference genome. Then the `MiClip.snp` function will take the alignment file as input. Because there is no real control sample from the original study, we use part of the test.sam file as a fake control sample for demonstration.

```
> test=MiClip.snp(test,file=system.file("extdata/snp.sam",package="MiClip"),mut.type="Del")
```

```
Identifying clusters finished!
Generating bin file finished!
```

4 Output of *MiClip*

4.1 Output format

`MiClip.binding` returns a `MiClip` object which normally comprises of three data frames.

```
> enriched=test$enriched # test will contain at least three data frames
> sites=test$sites
> clusters=test$clusters
> head(enriched) # view these data frames
```

```
  region_id   chr   start     end strand tag enriched probability
1         1 chr18 3341965 3341969      +   2    FALSE       1.000
2         1 chr18 3341970 3341974      +   3    FALSE       0.999
3         1 chr18 3341975 3341979      +   3    FALSE       0.999
4         1 chr18 3341980 3341984      +   3    FALSE       0.999
5         1 chr18 3341985 3341989      +   3    FALSE       0.999
6         1 chr18 3341990 3341994      +   3    FALSE       0.999
```

```
> head(sites)
```

```
  region_id sub_region_id strand   chr     pos tag mutant sites probability
1         7             1      + chr18 3399930   3      0 FALSE       0.989
2         7             1      + chr18 3399931   3      3  TRUE       0.996
3         7             1      + chr18 3399932   3      0 FALSE       0.995
4         7             1      + chr18 3399933   3      0 FALSE       0.999
5         7             1      + chr18 3399934  12      0 FALSE       1.000
6         7             1      + chr18 3399935  12      0 FALSE       1.000
    SNP
1 FALSE
2  TRUE
3 FALSE
4 FALSE
5 FALSE
6 FALSE
```

```
> head(clusters)
```

```
  region_id   chr strand   start     end enriched sites   SNP
1         1 chr18      + 3341965 3342004    FALSE FALSE FALSE
2         2 chr18      + 3362790 3362829    FALSE FALSE FALSE
3         3 chr18      + 3391135 3391169    FALSE FALSE FALSE
4         4 chr18      + 3395450 3395484    FALSE FALSE FALSE
5         5 chr18      + 3396420 3396454    FALSE FALSE FALSE
6         6 chr18      + 3399855 3399889    FALSE FALSE FALSE
```

```
> head(enriched[enriched$enriched,]) # view enriched bins
```

```
   region_id   chr   start     end strand tag enriched probability
49         7 chr18 3399930 3399934      +   5     TRUE       0.749
50         7 chr18 3399935 3399939      +  12     TRUE       1.000
51         7 chr18 3399940 3399944      +  12     TRUE       1.000
52         7 chr18 3399945 3399949      +  11     TRUE       1.000
53         7 chr18 3399950 3399954      +   9     TRUE       1.000
54         7 chr18 3399955 3399959      +   8     TRUE       1.000
```

```
> head(sites[sites$sites,]) # view binding sites
```

```
     region_id sub_region_id strand   chr     pos tag mutant sites probability
2            7             1      + chr18 3399931   3      3  TRUE       0.996
396         82            12      + chr18 4673570   7      3  TRUE       0.973
699        101            21      + chr18 4681128   7      2  TRUE       0.677
910        107            26      + chr18 4682164  14      4  TRUE       0.952
1021       111            28      + chr18 4682610  12      7  TRUE       1.000
1525       218            43      + chr18 5650699   6      3  TRUE       0.983
       SNP
2     TRUE
396  FALSE
699  FALSE
910  FALSE
1021  TRUE
1525 FALSE
```

```
> head(clusters[clusters$enriched,]) # view clusters with enriched bins
```

```
   region_id   chr strand   start     end enriched sites   SNP
7          7 chr18      + 3399910 3399969     TRUE  TRUE  TRUE
9          9 chr18      + 3405685 3405769     TRUE FALSE FALSE
15        15 chr18      + 3421220 3421359     TRUE FALSE FALSE
23        23 chr18      + 3426755 3426809     TRUE FALSE FALSE
27        27 chr18      + 3430955 3430994     TRUE FALSE FALSE
36        36 chr18      + 3435015 3435064     TRUE FALSE FALSE
```

```
> head(clusters[clusters$sites,]) # view clusters with binding sites
```

```
    region_id   chr strand   start     end enriched sites   SNP
7           7 chr18      + 3399910 3399969     TRUE  TRUE  TRUE
82         82 chr18      + 4673520 4673639     TRUE  TRUE FALSE
101       101 chr18      + 4681105 4681189     TRUE  TRUE FALSE
```

```
107        107 chr18      + 4681985 4682249     TRUE  TRUE FALSE
111        111 chr18      + 4682585 4682639     TRUE  TRUE  TRUE
218        218 chr18      + 5650685 5650729     TRUE  TRUE FALSE
```

`enriched` is the output of the first Hidden Markov Model. `region_id` is the id number for each cluster. `chr`, `strand`, `start` and `end` specify the genomic location of each bin. `tag` is the rounded average tag count in each bin. `enriched` and `probability` are the inference results.

`sites` is the output of the second Hidden Markov Model. `region_id` is the id number for the cluster which each base resides in. `sub_region_id` is the id number of the concatenated segment. Sometimes one enriched cluster has multiple modes, so it may be cut into two or more segments. `chr`, `strand` and `pos` specify the genomic location of each base. `tag` is the tag count and `mutant` is the mutant count on each base. `sites` and `probability` are the inference results.

`clusters` is the summary of results for all clusters. `region_id` is the id number for each cluster. `chr`, `strand`, `start` and `end` specify the genomic range. `enriched` specifies whether a cluster is found to have at least one enriched bin, and `sites` specifies whether a cluster is found to have at least one significant binding site.

4.2 SNPs

If we further process the `MiClip` object with `MiClip.snp`, a data frame `snps` will be added to the `MiClip` object. It contains information of the possible SNP sites extracted from the control experiment file. Also, a column will be added to `sites` specifying whether a binding site could actually be a SNP and another column will be added to `clusters` specifying whether a cluster contains a least one binding site which could actually be a SNP.

```
> snps=test$snps
> head(snps) # Inferred possible SNP sites are contained in this data frame

       chr strand     pos tag mutant
242  chr18      + 3399931   3      3
2314 chr18      + 4383748   3      3
2695 chr18      + 4394004   4      4
3939 chr18      + 4674843   3      3
5811 chr18      + 4682610  12      7
6153 chr18      + 4948777   4      4

> head(sites[sites$SNP,]) # In this dataset, three possible SNPs are found

     region_id sub_region_id strand   chr     pos tag mutant sites probability
2            7             1      + chr18 3399931   3      3  TRUE       0.996
1021       111            28      + chr18 4682610  12      7  TRUE       1.000
5146       745           139      - chr18 6227630   6      4  TRUE       0.999
      SNP
2    TRUE
1021 TRUE
5146 TRUE

> head(clusters[clusters$SNP,])
```

```
    region_id   chr strand   start     end enriched sites  SNP
7           7 chr18      + 3399910 3399969     TRUE  TRUE TRUE
111       111 chr18      + 4682585 4682639     TRUE  TRUE TRUE
745       745 chr18      - 6227590 6227664     TRUE  TRUE TRUE
```

4.3 MiClip.sum

MiClip provides a summary function `MiClip.sum`. Because we are using a very small toy sample data, the results presented are not realistic.

```
> MiClip.sum(test)

For identifying enriched regions
# of clusters: 1110
# of identified enriched clusters: 170
# of bins: 10624
# of bins in each state:
FALSE  TRUE
 9334  1290
Statistics of probability
   Min. 1st Qu.  Median    Mean 3rd Qu.    Max.
 0.2590  0.9990  1.0000  0.9763  1.0000  1.0000
Average tag count of enriched bin: 8
Average tag count of not enriched bin: 2


For identifying binding sites
# of enriched clusters: 170
# of sub enriched clusters: 179
# of enriched clusters with identified binding sites: 22
# of bases: 6365
# of bases in each state:
FALSE  TRUE
 6342    23
Statistics of probability
   Min. 1st Qu.  Median    Mean 3rd Qu.    Max.
 0.5380  1.0000  1.0000  0.9991  1.0000  1.0000
Average tag count of binding site: 7
Average mutant count of binding site: 3
Average tag count of not binding site: 8
Average mutant count of not binding site: 0
```

`MiClip.sum` gives the basic statistics on the results of the two rounds of Hidden Markov Model.

5 Session Info

```
> sessionInfo()
```

```
R version 2.15.0 (2012-03-30)
Platform: x86_64-redhat-linux-gnu (64-bit)

locale:
 [1] LC_CTYPE=en_US.UTF-8       LC_NUMERIC=C
 [3] LC_TIME=en_US.UTF-8        LC_COLLATE=C
 [5] LC_MONETARY=en_US.UTF-8    LC_MESSAGES=en_US.UTF-8
 [7] LC_PAPER=C                 LC_NAME=C
 [9] LC_ADDRESS=C               LC_TELEPHONE=C
[11] LC_MEASUREMENT=en_US.UTF-8 LC_IDENTIFICATION=C

attached base packages:
[1] stats4    splines   stats     graphics  grDevices utils     datasets
[8] methods   base

other attached packages:
[1] MiClip_1.0   VGAM_0.9-0   moments_0.13

loaded via a namespace (and not attached):
[1] tools_2.15.0
```

References

[1] Macias, S., et al. (2012) DGCR8 HITS-CLIP reveals novel functions for the Microprocessor. *Nat Struct Mol Biol* 19(8): p. 760-6.

[2] Chi SW, Zang JB, Mele A, Darnell RB. (2009) Argonaute HITS-CLIP decodes microRNA-mRNA interaction maps. *Nature* 2009 Jul 23;460(7254):479-86. Epub 2009 Jun 17.

[3] Lohse M, Bolger AM, Nagel A, Fernie AR, Lunn JE, Stitt M, Usadel B. (2012) RobiNA: a user-friendly, integrated software solution for RNA-Seq-based transcriptomics. *Nucleic Acids Res* 2012 Jul;40(Web Server issue):W622-7.
